# Efficacy and safety of four-year ofatumumab treatment in relapsing multiple sclerosis: The ALITHIOS open-label extension

**DOI:** 10.1177/13524585231195346

**Published:** 2023-09-11

**Authors:** Stephen L Hauser, Ronald Zielman, Ayan Das Gupta, Jing Xi, Dee Stoneman, Goeril Karlsson, Derrick Robertson, Jeffrey A Cohen, Ludwig Kappos

**Affiliations:** UCSF Weill Institute for Neurosciences and Department of Neurology, University of California, San Francisco (UCSF), 1651 4th Street, Box 3126, San Francisco, CA 94143, USA; Clinical Development, Novartis Pharma B.V., Amsterdam, The Netherlands; Analytics, Novartis Healthcare Pvt. Ltd., Hyderabad, India; China Novartis Institutes for Biomedical Research Co. Ltd., Novartis, Shanghai, People’s Republic of China; Global Medical, Novartis Pharma AG, Basel, Switzerland; Global Medical, Novartis Pharma AG, Basel, Switzerland; Department of Neurology, Multiple Sclerosis Division, Morsani College of Medicine, University of South Florida, Tampa, FL, USA; Department of Neurology, Mellen Center for MS Treatment and Research, Cleveland Clinic, Cleveland, OH, USA; MS Center and Research Center for Clinical Neuroimmunology and Neuroscience Basel (RC2NB), Department of Head, Spine and Neuromedicine, Biomedical and Clinical Research, University Hospital Basel, University of Basel, Basel, Switzerland

**Keywords:** Relapsing-remitting multiple sclerosis, ofatumumab, monoclonal antibodies, treatment, follow-up

## Abstract

**Background:**

Ofatumumab has demonstrated superior efficacy and favorable safety for up to 2.5 years versus teriflunomide in relapsing multiple sclerosis (RMS).

**Objective:**

Further characterize efficacy and safety of ofatumumab in RMS.

**Methods:**

Efficacy set: patients randomized to ofatumumab/teriflunomide in ASCLEPIOS I/II (core). Safety set: patients who received ⩾ 1 dose of ofatumumab in ASCLEPIOS I/II, APLIOS, APOLITOS (all core), or ALITHIOS (umbrella open-label extension). Patients received continuous ofatumumab or were newly switched from teriflunomide. Data cut-off: 25 September 2021.

**Results:**

In the efficacy set (*n* = 1882), the continuous ofatumumab group had a low annualized relapse rate (ARR 0.05 (95% confidence interval: 0.04–0.07)), low numbers of gadolinium-enhancing (Gd+) T1 lesions (0.01 lesions/scan) and fewer new/enlarging T2 lesions (annualized rate 0.08). Overall, 78.8% met three-parameter “no evidence of disease activity” criteria through 4 years. Switching from teriflunomide led to reduced ARR, risk of confirmed disability worsening (CDW), new/enlarging T2 lesions, Gd+ T1 lesions, and serum neurofilament light chain. In the continuous and newly switched ofatumumab groups, cumulative 3- and 6-month CDW rates remained low. In the safety set (*n* = 1969), the most frequently reported adverse events were infections and infestations (58.35%). No new safety signals were identified.

**Conclusion:**

Ofatumumab has a favorable longer-term benefit–risk profile in RMS.

**Trial registry:**

ALITHIOS (NCT03650114): https://clinicaltrials.gov/ct2/show/NCT03650114

## Introduction

Ofatumumab is a fully human, anti-CD20 monoclonal antibody (mAb) approved for relapsing multiple sclerosis (RMS) in many countries.^1,2^ The approved regimen with three weekly and then monthly (Q4W) subcutaneous (s.c.) ofatumumab 20 mg produces a rapid, sustained B-cell depletion, with minimal repletion between doses.^3^

Ofatumumab approval was based on the ASCLEPIOS I (NCT02792218) and ASCLEPIOS II (NCT02792231) Phase 3 studies, which demonstrated superior efficacy versus teriflunomide and a favorable benefit–risk profile up to 2.5 years.^4,5^ Ofatumumab achieved the primary endpoint, a reduction in annualized relapse rate (ARR); 51% in ASCLEPIOS I and 58% in ASCLEPIOS II (both *p* < 0.001), and demonstrated greater efficacy versus teriflunomide for most secondary clinical and magnetic resonance imaging (MRI) outcomes.^4^

To further assess the benefit–risk profile of ofatumumab in RMS and its longer-term tolerability, patients from ofatumumab studies (ASCLEPIOS I/II,^4^ APLIOS,^6^ and APOLITOS)^7^ transitioned to ALITHIOS (NCT03650114), a Phase 3b, open-label, extension, where they continued with ofatumumab or switched from placebo/teriflunomide to ofatumumab. After 3.5 years of follow-up in ALITHIOS, ofatumumab was well tolerated with no new safety signals; rates of adverse events (AEs) and serious AEs (SAEs) were consistent with previous findings.^8^

Emerging data indicate that early initiation of high-efficacy therapies for RMS improves longer-term outcomes compared with delayed initiation of, or escalation from lower efficacy therapies.^9–11^ A subgroup analysis of recently diagnosed, treatment-naïve patients in ASCLEPIOS I/II concluded that ofatumumab has a favorable benefit–risk profile versus teriflunomide,^12^ supporting first-line use of ofatumumab in these patients.

We report the efficacy and safety of ofatumumab in patients with RMS, for up to 4 years.

## Patients and methods

### Trial design and patients

**Figure 1.**
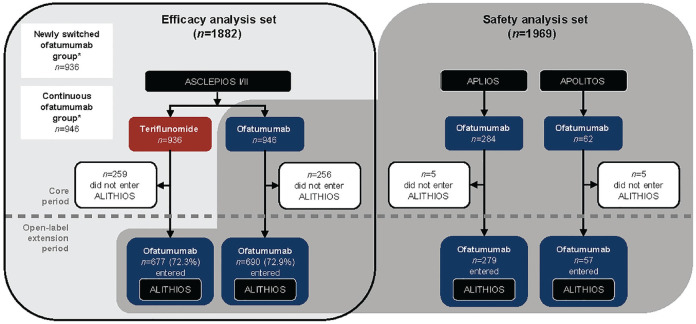
Analysis sets and time periods. *The “newly switched ofatumumab group” comprised patients randomized to teriflunomide in ASCLEPIOS I/II (those who completed treatment were eligible to enter ALITHIOS and be switched to ofatumumab); the “continuous ofatumumab group” comprised patients randomized to ofatumumab in ASCLEPIOS I/II (those who completed treatment were eligible to enter ALITHIOS). All patients who completed study treatment in the core period were eligible to enter ALITHIOS but could withdraw prior to treatment. The efficacy analysis set comprised all patients randomized to ofatumumab or teriflunomide in ASCLEPIOS I/II, regardless of whether they completed/discontinued study treatment. The safety analysis set comprised patients who received ≥1 dose of ofatumumab in ASCLEPIOS I/II, APLIOS, APOLITOS, or ALITHIOS. Percentages based on the number of randomized patients. The open-label extension period was defined as following the first dose of ofatumumab in ALITHIOS (below the dashed line); the core period is prior to the open-label extension (above the dashed line). Reasons for the discontinuation of study treatment are presented in Supplementary Table 1.

The methods of ASCLEPIOS I/II, APLIOS, and APOLITOS have been reported previously.^4,6,7^ ALITHIOS is an ongoing, Phase 3b, open-label, umbrella extension study (initiated 22 November 2018) to assess the longer-term safety, tolerability, and effectiveness of ofatumumab (20 mg s.c. Q4W) in patients with RMS.^8^ Patients who completed treatment in the core periods of ASCLEPIOS I/II, APLIOS,^6^ or APOLITOS^7^ could enter ALITHIOS (see Figure 1). For key inclusion/exclusion criteria, refer to Supplementary Materials. Interim analyses presented here are from the core and extension periods (data cut-off (DCO): 25 September 2021).

### Analysis populations

#### Efficacy analysis set

Efficacy analyses consisted of data from patients randomized to ofatumumab or teriflunomide in ASCLEPIOS I/II. For three-parameter no evidence of disease activity (NEDA-3), a modified efficacy analysis set was used, excluding patients who discontinued treatment early except due to lack of efficacy or death and had NEDA-3 prior to early discontinuation.

#### Safety analysis set

Safety analyses consisted of data from patients who received ⩾ 1 dose of ofatumumab in ASCLEPIOS I/II, APLIOS, APOLITOS, or ALITHIOS (see Figure 1).

#### Analysis of delayed versus early initiation of ofatumumab

Two analysis subgroups were defined: the “continuous ofatumumab group” and the “newly switched ofatumumab group” (see Figure 1). Continuous ofatumumab group: in the efficacy analysis, this comprised patients randomized to ofatumumab in ASCLEPIOS I/II, whereas in the safety analysis, this comprised patients who received ⩾ 1 dose of ofatumumab in ASCLEPIOS I/II, APLIOS, or APOLITOS. Newly switched ofatumumab group: in both the efficacy and safety analyses, this comprised patients who received teriflunomide in ASCLEPIOS I/II and switched to ofatumumab in ALITHIOS.

### Efficacy endpoints

The endpoints assessed were: ARR; confirmed disability worsening (CDW) events (increase from baseline Expanded Disability Status Scale score sustained for ⩾ 3/6 months (3/6mCDW) mean number of gadolinium-enhancing (Gd+) T1 lesions per scan; number of new/enlarging T2 (neT2) lesions per year; serum neurofilament light chain (sNfL) concentration at ASCLEPIOS I/II baseline, 3-/12 months post-baseline, and every 6 months thereafter; and NEDA-3 status (no 6mCDW events, no confirmed relapses, and no MRI activity (new Gd+ T1 or neT2 lesions)) in the core and extension periods, in Year 1, beyond Year 1, and overall. For further details, see Supplementary Materials.

### Safety and tolerability evaluation

AEs were graded according to the Common Terminology Criteria for Adverse Events version 5.0,^13^ with preferred terms per Medical Dictionary for Regulatory Activities version 24.1. An independent expert reviewed cases of opportunistic infection.

### Statistical analyses

The ARR for the newly switched and continuous ofatumumab groups was estimated using a piecewise negative binomial model. Cumulative 3/6mCDW were assessed using Kaplan–Meier curves. Lesions per scan and adjusted annualized rates of lesions were estimated using piecewise negative binomial models. Between-group comparisons for number of relapses and number of lesions were analyzed using the Wilcoxon rank sum test. sNfL concentration was analyzed by mixed-effect modeling of repeated measures. NEDA-3 during the core and extension periods, and overall was analyzed separately using logistic regression models fitted to modified efficacy analysis sets, which excluded patients who discontinued early for reasons other than lack of efficacy/death and had NEDA-3 prior to discontinuation.

## Results

### Patients

#### Efficacy subgroups: baseline demographics and reasons for discontinuation

The efficacy analysis set included 1882 patients: in ASCLEPIOS I/II, 936 and 946 patients were randomized to teriflunomide and ofatumumab, respectively; 72.6% (*n* = 677 (teriflunomide) *n* = 690 (ofatumumab) entered ALITHIOS (see Figure 1). In total, *n* = 1214/1367 (88.8%) were receiving ofatumumab at DCO. Baseline demographics and disease characteristics were balanced between groups (see Table 1). In ALITHIOS, the most common reasons for ofatumumab discontinuation were AEs and patient/guardian decision (both ~4%, see Supplementary Table 1).

**Table 1.** Baseline demographics and clinical characteristics (efficacy analysis set^a^).

	Continuous ofatumumab group (*N* = 946)	Newly switched ofatumumab group^a^	
		Baseline of core period (*N* = 936)	Baseline of open-label extension period (*N* = 677)
Age, years (mean ± SD)	38.4 ± 9.04	38.0 ± 9.22	40.1 ± 9.21
Age group, *n* (%) years			
18 to 30	223 (23.6)	219 (23.4)	116 (17.1)
31 to 40	306 (32.3)	345 (36.9)	239 (35.3)
41 to 55	414 (43.8)	371 (39.6)	288 (42.5)
> 55	3 (0.3)	1 (0.1)	34 (5.0)
Female, *n* (%)	637 (67.3)	636 (67.9)	456 (67.4)
BMI, kg/m^2^ (mean ± SD)	25.86 ± 6.22	25.93 ± 6.02	25.61 ± 5.85
Treatment-naive patients^b^, *n* (%)	386 (40.8)	363 (38.8)	Not applicable^c^
EDSS score at baseline (mean ± SD)	2.93 ± 1.35	2.90 ± 1.36	2.81 ± 1.46^d^
Number of relapses in the last 12 months prior to screening (mean ± SD)	1.2 ± 0.69	1.3 ± 0.71	0.2 ± 0.49^d^
Number of Gd+ T1 lesions (mean ± SD)	1.7 ± 4.51	1.3 ± 3.43	0.8 ± 2.37^d^
Total volume of T2 lesions, cm^3^ (mean ± SD)	13.72 ± 13.80	12.55 ± 13.81	Not available
Type of MS at study entry, *n* (%)			
RRMS	890 (94.1)	884 (94.4)	
SPMS	56 (5.9)	52 (5.6)	
Time since first MS symptom, years (mean ± SD)	8.27 ± 7.13	8.19 ± 7.29	9.94 ± 7.23
Time since MS diagnosis, years (mean ± SD)	5.68 ± 6.21	5.56 ± 6.10	7.33 ± 6.02

BMI: body mass index; EDSS: Expanded Disability Status Scale; Gd+: gadolinium-enhancing; MS: multiple sclerosis; RRMS: relapsing remitting multiple sclerosis; SD: standard deviation; SPMS: secondary progressive multiple sclerosis.

Data from the efficacy analysis set.

a Patients who completed treatment with teriflunomide in the core period were switched to ofatumumab in the open-label extension period.

b Treatment-naive patients had not received a prior multiple sclerosis disease modifying therapy.

c Not applicable, as all patients had previously received teriflunomide.

d Values at the baseline of the open-label extension period in the newly switched ofatumumab group reflect the teriflunomide treatment effect during the core period.

#### Safety analyses

The safety analysis set included 1969 patients (see Figure 1). Baseline demographics and disease characteristics of the continuous ofatumumab (*n* = 1292/1969) and newly switched ofatumumab group (*n* = 677/1969) were comparable (see Supplementary Table 2). Total ofatumumab exposure was 4032.5 patient-years (PYs; continuous ofatumumab 2761.4 versus newly switched ofatumumab 1271.1 PYs), and mean ofatumumab exposure was 2.9 and 1.9 years, respectively (see Supplementary Table 3). Mean adherence to ofatumumab treatment was > 95% (see Supplementary Table 4).

### Clinical efficacy assessments

#### Relapses

**Figure 2.**
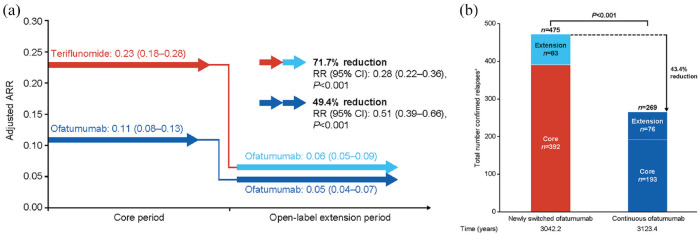
Relapse data: (a) within-group comparison of ARR during the core and open-label extension periods; (b) between-group comparison of total confirmed relapses up to 4 years in the newly switched ofatumumab group and continuous ofatumumab group (efficacy analysis set). Data from the efficacy analysis set. Adjusted ARR presented as ARR (95% CI). ^a^Confirmed relapses are those accompanied by a clinically relevant change in the EDSS. Comparisons obtained from fitting a piecewise negative binomial model for the core period and open-label extension time periods with log-link, adjusted for treatment and region as factors, number of relapses in previous year, baseline EDSS score, baseline number of Gd+ lesions and the patient’s age at baseline as covariates. The natural log of the time-in-study (in years) by time period is used as offset to annualize the relapse rate in each time period. Baseline variables are from the core period baseline. *P* values in panel A are nominal *P* values; *P* value in panel B is from a Wilcoxon Rank Sum test. ARR: annualized relapse rate; CI: confidence interval; EDSS, Expanded Disability Status Scale; Gd+, gadolinium-enhancing; RR: rate ratio.

The continuous ofatumumab group maintained a low ARR for up to 4 years: adjusted ARRs in the core and extension periods were 0.11 (95% confidence interval (CI): 0.08–0.13) and 0.05 (95% CI: 0.04–0.07), respectively (49.4%; *p* < 0.001), corresponding to an adjusted rate of one relapse every 20 years during the extension period. For the newly switched ofatumumab group, the ARR was reduced by 71.7% in the extension versus core period (*p* < 0.001); adjusted ARRs were 0.23 (95% CI: 0.18–0.28) and 0.06 (95% CI: 0.05–0.09), respectively (see Figure 2(a)). See Supplementary Figure 1A for between-group comparison. The cumulative number of confirmed relapses in the continuous ofatumumab group (*n* = 269; 3123.4 years) was 43.4% lower (between-group analysis; *p* < 0.001) than in the newly switched ofatumumab group (*n* = 475; 3042.2 years; see Figure 2(b)).

#### CDW events

**Figure 3.**
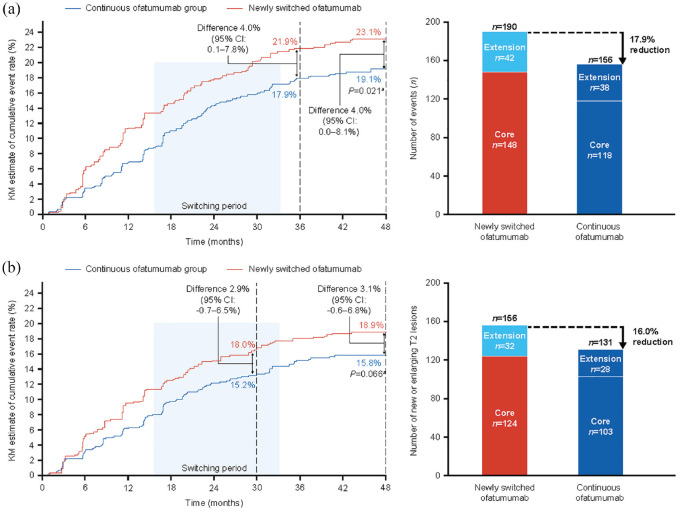
Kaplan–Meier estimates of cumulative event rates (% patients) for confirmed disability worsening (left-hand panel) and cumulative number of confirmed disability worsening events (right-hand panel): (a) 3mCDW and (b) 6mCDW (efficacy analysis set). Data from the efficacy analysis set. Superior efficacy of ofatumumab in the core period was established previously versus teriflunomide (for more information, refer to Hauser, et al.^4^). Cut-off for core and open-label extension periods based on the first dose of ofatumumab in the open-label extension period. “Difference” refers to the difference in KM estimates (newly-switched ofatumumab group minus continuous ofatumumab group). ^a^*P* value is a Log-Rank test. 3mCDW: 3-month confirmed disability worsening; 6mCDW: 6-month confirmed disability worsening; CI: confidence interval; HR, hazard ratio; KM: Kaplan–Meier.

Cumulative 3/6mCDW event rates (Kaplan–Meier estimate) remained lower with continuous ofatumumab versus newly switched ofatumumab (3mCDW rate at Month 48: 19.1% and 23.1%, respectively; overall log-rank *p* = 0.021, see Figure 3(a); 6mCDW rate at Month 48: 15.8% and 18.9%, respectively; overall log-rank *p* = 0.066, see Figure 3(b)). Cumulative numbers of 3mCDW and 6mCDW events were 17.9% and 16.0% lower in the continuous ofatumumab group versus the newly switched ofatumumab group (3mCDW events: *n* = 156 versus *n* = 190, respectively, see Figure 3(a); 6mCDW events: *n* = 131 versus *n* = 156, see Figure 3(b)).

#### MRI assessments

**Figure 4.**
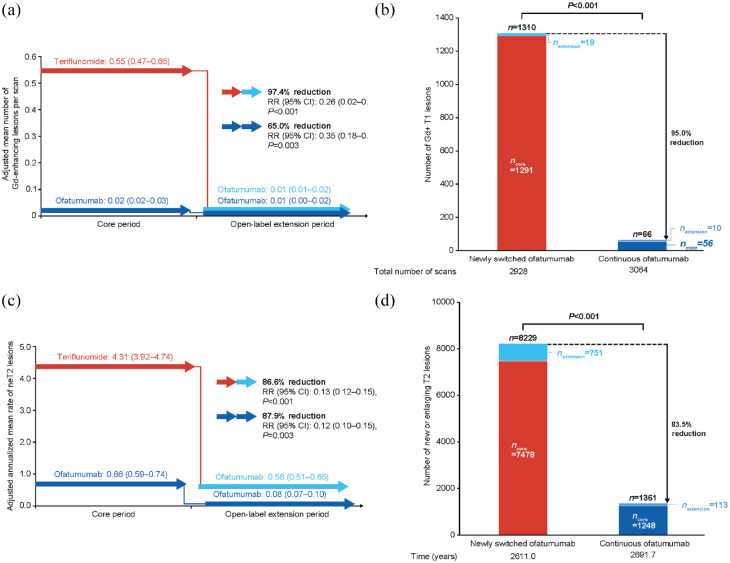
Lesion activity on MRI in the core and extension periods: (a) within-group comparison, mean number of Gd+ T1 lesions/scan; (b) between-group comparison, mean number of Gd+ T1 lesions; (c) within-group comparison, mean annualized rate of neT2 lesions; and (d) between-group comparison, mean number of neT2 lesions (efficacy analysis set). Data from the efficacy analysis set. Panels A and C: data are presented as mean (95% CI). Data were estimated from fitting a piecewise negative binomial model for the core and open-label extension time periods with log-link, adjusted for treatment and region as factors for panels A and B, and treatment for panels C and D. For panels A and B, baseline number of T1 Gd+ lesions and patient’s age at baseline were covariates; the natural log of the number of scans with evaluable Gd+ lesion counts by period is used as offset to obtain the lesion rate per scan in each period. For panels C and D, baseline volume of T2 lesions and patient’s age at baseline were covariates; the natural log of the time-in-study (in years) by period is used as offset to annualize the lesion rate in each period. Baseline variables are from the core period baseline. *P* values in panels A and C are nominal *P* values; *P* values in panels B and D are from a Wilcoxon Rank Sum test. CI: confidence interval; Gd+: gadolinium-enhancing; neT2: new/enlarging T2; RR, rate ratio.

Near-complete suppression of MRI activity was maintained through 4 years in the continuous ofatumumab group. The adjusted mean number of Gd+ T1 lesions per scan in the core and extension periods was: 0.02 (95% CI: 0.02–0.03) and 0.01 (95% CI: 0.00–0.02), respectively; a reduction of 65.0% (*p* = 0.003). For the newly switched ofatumumab group, the adjusted mean number of Gd+ T1 lesions per scan reduced from 0.55 (95% CI: 0.47–0.65) in the core period, to 0.01 (95% CI: 0.01–0.02) in the extension period; a reduction of 97.4% (*p* < 0.001; see Figure 4(a)). The cumulative number of Gd+ T1 lesions in the continuous ofatumumab group (*n* = 66) was 95% lower (*p* < 0.001) versus the newly switched ofatumumab group (*n* = 1310; see Figure 4(b)). For between-group comparison, see Supplementary Figure 1B.

Continuous ofatumumab treatment led to an 87.9% reduction (*p* < 0.001) in the adjusted annualized rate of neT2 lesions (core period: 0.66 (95% CI: 0.59–0.74); extension period: 0.08 (95% CI 0.07–0.10)). There was also a reduction with the newly switched ofatumumab group, where the adjusted mean number of neT2 lesions per scan decreased from 4.31 (95% CI: 3.92–4.74) in the core period, to 0.58 (95% CI: 0.51–0.65) in the extension period; an 86.6% per scan rate reduction (*p* < 0.001; see Figure 4(c)). The cumulative number of neT2 lesions in the continuous ofatumumab group (*n* = 1361 at DCO) was reduced by 83.5% (*p* < 0.001) versus the newly switched ofatumumab group (*n* = 8229; see Figure 4(d)). For between-group comparison of the annualized rate of neT2 lesions, see Supplementary Figure 1C.

#### sNfL concentration

In the core period, sNfL concentration was lower with ofatumumab versus teriflunomide (concentration at Month 12: 8.03 versus 10.25 pg/mL; at Month 24: 7.96 versus 9.97 pg/mL, respectively; both *p* < 0.001; Supplementary Figure 2A). sNfL concentration remained low in the extension period with continuous ofatumumab treatment (Month 24: 8.50 pg/mL; Supplementary Figure 2B). Switching from teriflunomide to ofatumumab lowered sNfL levels: in the newly switched ofatumumab group, sNfL concentration remained higher versus the continuous ofatumumab group up to 6 months after switch (9.07 versus 8.31 pg/mL; *p* < 0.001), but low in both groups at 24 months (8.23 versus 8.50 pg/mL; see Supplementary Figure 2B). The benefits of continuous ofatumumab treatment on sNfL concentration were evident for up to 48 months (see Supplementary Figure 2C).

#### NEDA-3 status

**Figure 5.**
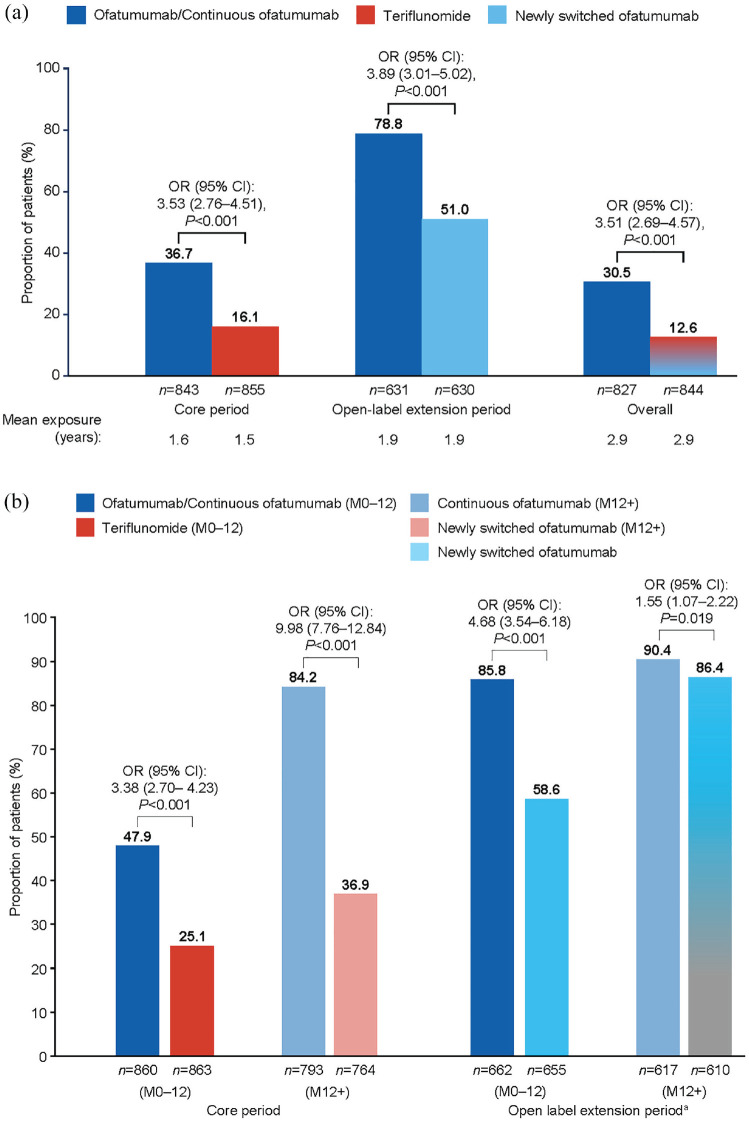
No evidence of disease activity (NEDA-3): (a) core period, open-label extension period and overall; (b) during the first year of treatment and after first year of treatment, by study period (efficacy analysis set). Data from the modified efficacy analysis set for NEDA-3. All *P* values are nominal. The statistical model used logistic regression to adjust for treatment and region as factors, and for age, baseline EDSS score, number of Gd+ lesions at baseline as covariates. N=total patients in each treatment group. ^a^Re-baseline was performed at entry to the extension period. CI: confidence interval; EDSS, Expanded Disablity Status Scale; Gd+, gadolinium-enhancing; M: month; NEDA-3: three-parameter no evidence of disease activity; OR: odds ratio.

The likelihood of maintaining NEDA-3 for up to 4 years was over three times higher with early ofatumumab initiation. A greater proportion of the continuous ofatumumab group than the newly switched ofatumumab group maintained NEDA-3 during the core period: 36.7 versus 16.1% (odds ratio (OR) 3.53 (95% CI: 2.76–4.51), *p* < 0.001); extension period: 78.8 versus 51.0% (OR 3.89 (95% CI: 3.01–5.02), *p* < 0.001); and overall: 30.5 versus 12.6% (OR 3.51 (95% CI: 2.69–4.57, *p* < 0.001; see Figure 5(a)). See Supplementary Figures 3A–D for data on each component of NEDA-3.

To explore the influence of rapid suppression of inflammatory activity on MRI with ofatumumab, NEDA-3 status in the first year of treatment was determined (see Figure 5(b)). In the core period, the likelihood of maintaining NEDA-3 during the first year was approximately three-fold higher with ofatumumab versus teriflunomide (47.9 versus 25.1% OR 3.38 (95% CI: 2.70–4.23) *p* < 0.001), and 10-fold higher beyond that time (84.2 versus 36.9%; OR 9.98 (95% CI: 7.76–12.84), *p* < 0.001; see Figure 5(b)). In the extension period, 85.8% of the continuous ofatumumab group and 58.6% of the newly switched ofatumumab group maintained NEDA-3 during the first year (OR 4.68 (95% CI: 3.54–6.18), *p* < 0.001). Subsequently, NEDA-3 increased to 86.4% in the newly switched ofatumumab group and became comparable to the proportion in the continuous ofatumumab group (90.4%; OR 1.55 (95% CI: 1.07–2.22); *p* = 0.019).

### Safety

#### AE profile

Safety was consistent with previous reports^4,8^ and no new safety signals emerged. Overall, *n* = 1698/1969 (86.23%) of the ofatumumab safety analysis set had ⩾ 1 AE; the exposure-adjusted incidence rate (EAIR) was 135.11 per 100 PYs (95% CI: 128.83–141.69; see Table 2). In the ASCLEPIOS I/II core period, *n* = 791/946 (83.61%) of the ofatumumab group had ⩾ 1 AE and the EAIR was 188.55 (95% CI: 175.86–202.16). The incidence of AEs leading to discontinuation was low (overall ofatumumab safety analysis set: *n* = 128/1969 (6.50%); ASCLEPIOS I/II core period: *n* = 54/946 (5.70%)). Serious AEs were reported in *n* = 242/1969 (12.30%) of the ofatumumab safety analysis set (EAIR 4.96 (95% CI: 4.37–5.63)), and in *n* = 86/946 (9.10%) of the ofatumumab group in the core period (EAIR 5.39 (95% CI: 4.36–6.65)). The most frequently reported AEs were infections and infestations (overall ofatumumab safety analysis set: *n* = 1149/1969 (58.35%); ACLEPIOS I/II core period: *n* = 488/946 (51.58%)) consistent with previous findings up to 2.5 years (*n* = 488/946 (51.6%).^4,5^

**Table 2.** Safety summary (safety analysis set).

Adverse event	ASCLEPIOS I/II core period, ofatumumab group (*N* = 946)^a^		Overall ofatumumab (*N* = 1969)^b^	
	*n* (%)	EAIR [95% CI]	*n* (%)	EAIR [95% CI]
Patients with at least one AE	791 (83.61)	188.55 [175.86–202.16]	1698 (86.23)	135.11 [128.83–141.69]
Patients with at least one SAE	86 (9.10)	5.39 [4.36–6.65]	242 (12.30)	4.96 [4.37–5.63]
AEs leading to ofatumumab discontinuation	54 (5.70)	0	128 (6.50)	0
Infections and infestations	488 (51.58)	51.14 [46.80–55.88]	1149 (58.35)	40.95 [38.65–43.39]
Serious infections	24 (2.54)	1.44 [0.97–2.15]	78 (4.01)	1.53 [1.23–1.91]
Injection-related systemic reactions	195 (20.61)	15.49 [13.46–17.83]	487 (24.73)	12.38 [11.33–13.53]
Injection site reactions	103 (10.88)	7.21 [5.94–8.74]	233 (11.83)	5.00 [4.40–5.68]
Malignancies	5 (0.53)	0.32 [0.13–0.77]	17 (0.86)	0.33 [0.20–0.53]
Deaths	0	0	6^c^ (0.30)	0

AE: adverse event; CI: confidence interval; EAIR: exposure adjusted incidence rate per 100 patient years; SAE: serious adverse event.

Preferred terms are according to MedDRA version 24.1.

Data from the safety analysis set.

a Data are from the core period.

b Data from both the core and open-label extension periods.

c Causes of death were: sudden death (*n* = 1); suicide (*n* = 1); COVID-19 and COVID-19 pneumonia (*n* = 1); COVID-19 (*n* = 1); intestinal metastasis (*n* = 1); pneumonia and septic shock (*n* = 1).

#### Serious infections

The EAIR of serious infections remained stable (overall safety analysis set (*n* = 78/1969) 1.53 (95% CI: 1.23–1.91); ASCLEPIOS I/II core period ofatumumab group (*n* = 24/946) 1.44 (95% CI: 0.97–2.15); see Table 2). The most frequent were COVID-19 infections (*n* = 1/1969; 0.05%) and appendicitis (*n* = 14/1969; 0.7%). Most serious infections (*n* = 73/1969; 3.7%) resolved without discontinuation. A small proportion of serious infections (*n* = 6/1969; 0.3%) were Grade 4.

#### Immunoglobin G and immunoglobin M

Mean serum immunoglobin G (IgG) levels remained stable and above the lower limit of normal (LLN; 5.65 g/L), even in patients with lower IgG levels at baseline (see Supplementary Figures 5A and 5C); mean serum immunoglobin M (IgM) levels decreased but remained above the LLN (0.40 g/L; see Supplementary Figure 5B and D). Of 1969 patients, interruption was reported in 2 (0.1%) and 185 (9.5%) patients due to low IgG and IgM, and discontinuation in 1 (0.1%) and 60 (3.0%) patients, respectively (see Supplementary Tables 6 and 7). Sensitivity analyses confirmed early interruption/discontinuation of ofatumumab due to low IgG/IgM did not impact overall IgG/IgM patterns (see Supplementary Figures 5E–5G). Low levels of IgG or IgM were not associated with an increased incidence of serious infections (see Supplementary Table 8).

#### Neutropenia and lymphopenia

Mean lymphocyte and neutrophil levels remained stable and were above the LLN; any reductions below the LLN occurred randomly and were not persistent (see Supplementary Figure 6A and 6B). During the core period, although neutrophil levels were lower with teriflunomide, they returned to baseline levels following the switch to ofatumumab (see Supplementary Figure 6B). No serious AEs of lymphopenia or neutropenia were reported. Treatment was interrupted in 2/1969 (0.1%) patients with lymphopenia; neither discontinued treatment. There was no association between decreased lymphocyte/neutrophil counts and risk of serious infection.

#### Injection-related reactions

Of 1969 patients, 487 (24.7%) and 233 (11.8%) experienced a systemic and injection site-related reaction, respectively. Most systemic and injection site-related reactions (IRRs) were Grade 1/2 (*n* = 483/487 (99.2%) and *n* = 232/233 (99.5%); see Supplementary Figure 4A and 4B). No life-threatening IRRs were reported.

#### Malignancies

The incidence of malignancies was low (overall safety analysis set: 0.86%; EAIR 0.33 (95% CI: 0.20–0.53), see Supplementary Table 5); with an EAIR comparable to the core period (ASCLEPIOS I/II ofatumumab group: 0.53%; EAIR 0.32 (0.13–0.77)).

#### Deaths

In total, 6/1969 deaths occurred during the extension period and were reported by investigators as unrelated to ofatumumab (see Table 2).

## Discussion

This interim analysis provides new insights into the longer-term efficacy and safety of ofatumumab in RMS, supporting a favorable benefit–risk profile. The low rate of relapses with ofatumumab, as identified in ASCLEPIOS I/II,^4^ was further reduced in the extension period, and together with the almost complete suppression of MRI lesions and low risk of CDW, demonstrate the sustained efficacy of ofatumumab for up to 4 years.

Early initiation of high-efficacy treatment for RMS improves longer-term outcomes compared with delayed initiation or switching from lower efficacy therapies.^9–11^ This comparison of continuous ofatumumab versus switching to ofatumumab from teriflunomide demonstrated the cumulative benefit (up to 4 years) of early ofatumumab initiation with regard to cumulative number of relapses, and number of Gd+ T1/neT2 lesions. Moreover, the odds of maintaining NEDA-3 during the extension period were three times greater with early initiation. After the first year in the core period, a very high proportion (84.2%) of the continuous ofatumumab group retained NEDA-3 status; this beneficial effect continued throughout the extension period, supporting the sustained efficacy of ofatumumab. In contrast, only 36.9% of patients randomized to teriflunomide retained NEDA-3 status after a year. Although between-group differences for all components of NEDA-3 favored continuous ofatumumab, the greatest differences were for neT2 and Gd+ T1 lesions. Thus, patients initially randomized to teriflunomide were at higher risk of not maintaining NEDA-3 in the first year of treatment mainly due to MRI disease activity. The legacy of lower efficacy teriflunomide appeared to negatively affect the likelihood of maintaining NEDA-3 during the first year of open-label ofatumumab treatment, but with longer treatment, the odds of maintaining NEDA-3 increased to 9/10 patients and remained high for up to 4 years.

The 3mCDW and 6mCDW rates at 36 and 48 months, and the cumulative number of events, indicate that early ofatumumab treatment leads to superior disability outcomes that cannot be recovered in patients initially randomized to teriflunomide. These findings are consistent with recent studies of the longer-term benefits of early initiation of high-efficacy DMTs and disability outcomes in MS.^9–11^

The results also illustrate the value of switching from a low efficacy therapy to ofatumumab. In the newly switched ofatumumab group, there was a marked reduction in the ARR, together with almost complete suppression of Gd+ T1 lesion activity, a pronounced reduction of neT2 lesions, sustained reduction of neuroaxonal injury (sNfL), and increased likelihood of maintaining NEDA-3 status.

Treatment with biological drugs may trigger an immune response that leads to the formation of anti-drug antibodies (ADAs).^14^ The development of high titers of neutralizing ADAs may lead to suboptimal treatment exposure and thereby might limit efficacy.^14^ However, it has been previously reported that very few patients receiving ofatumumab in ASCLEPIOS I/II developed ADAs (0.2%), with no treatment enhancing or neutralizing ADAs reported (0%).^1,15^

The cumulative safety data indicate that extended ofatumumab treatment is well tolerated in patients with RMS, with no new risks identified. Ofatumumab tolerability was reflected by a high level of adherence and a low rate of discontinuation throughout the core and extension periods. The EAIRs of AEs and SAEs were consistent with those previously reported in ASCLEPIOS I/II.^4^ During extended exposure, the incidence of malignancies remained low, and both serious infections (EAIRs) and IgG levels remained stable. Although IgM levels declined, average IgM levels remained above the LLN. Neither low IgG nor low IgM were associated with increased incidence of serious infection. IgG levels fell below the LLN in only 1.6% of patients, and in most cases, did not lead to treatment interruption/discontinuation. Additional sensitivity analyses confirmed that treatment interruption/discontinuation due to low IgG/IgM did not impact overall Ig trends confirming the robustness of IgG stabilization. With longer-term use of ofatumumab, lymphocyte and neutrophil levels remained stable and above the LLN. The incidence of lymphopenia and neutropenia were comparable with previous studies and remained low, with no serious events reported.^4,6,7^ Overall, safety was in line with a previous interim analysis with up to 3.5 years of treatment.^8^

The analyses of longer-term efficacy data presented here for the open-label ALITHIOS extension study (including data from the start of the blinded ASCLEPIOS I/II studies) are subject to the limitations of any open-label study, including lack of a comparator arm beyond the core studies. As such, the conclusions related to longer-term clinical efficacy outcomes may be limited due to the potential influence of regression to the mean. Also, as blinding is not maintained during an open-label extension study, the risk of rater assessment bias may increase.

In addition, upon completion of the core studies, participation in ALITHIOS was voluntary and patients were free to discontinue due to any reason, creating the potential for selection bias. This seems unlikely, however, as an equal proportion of patients from the ASCLEPIOS I/II teriflunomide (72.3%) and ofatumumab (72.9%) arms enrolled into ALITHIOS; 88.8% of whom were still receiving ofatumumab at data cut-off. Furthermore, in ALITHIOS, the rate of discontinuations was identical for both the ofatumumab and teriflunomide arms (11.2%) and reasons for discontinuation were similar (for example, discontinuations due to AEs: 4.2% vs 3.8%, respectively; patient/guardian decision: 3.6% vs 4.3%, respectively), with low rates of discontinuations due to lack of efficacy (0.6% vs 0.9%, respectively; see Supplementary Table 1). Thus, there was no evidence for an impact of selection bias on these data.

The ongoing ALITHIOS study coincided with the global outbreak of COVID-19. People with MS are at increased risk of serious infection,^16–19^ and anti-CD20 mAbs may compromise the immune response^20^ and attenuate humoral responses to COVID-19 vaccination.^21–23^ In this interim analysis, most reported COVID-19 cases were non-serious, and 241/245 (98.4%) of patients recovered.^24^ Two COVID-19-related deaths (2/245 (0.8%) occurred in unvaccinated ofatumumab-treated patients (one with additional risk factors); both were deemed unrelated to study treatment. Fatalities occurred less frequently than reported for the general population.^25^

Our findings of sustained reductions in cumulative number of relapses, MRI lesion activity, and risk of CDW with ofatumumab treatment add to a growing body of evidence that supports the value of early initiation of high-efficacy therapies in RMS,^4,10^ and highlight the favorable benefit–risk profile of long-term (up to 4 years) ofatumumab treatment for patients with RMS. Early access to highly efficacious treatments such as ofatumumab can help to reduce disease burden, risk of RMS progression, and contribute to longer-term improvements in quality of life.^26^

## Supplemental Material

sj-docx-1-msj-10.1177_13524585231195346 – Supplemental material for Efficacy and safety of four-year ofatumumab treatment in relapsing multiple sclerosis: The ALITHIOS open-label extension Click here for additional data file. Supplemental material, sj-docx-1-msj-10.1177_13524585231195346 for Efficacy and safety of four-year ofatumumab treatment in relapsing multiple sclerosis: The ALITHIOS open-label extension by Stephen L Hauser, Ronald Zielman, Ayan Das Gupta, Jing Xi, Dee Stoneman, Goeril Karlsson, Derrick Robertson, Jeffrey A Cohen and Ludwig Kappos in Multiple Sclerosis Journal

## References

[1] US Food and Drug Administration. KESIMPTA® (ofatumumab) prescribing information, https://www.novartis.us/sites/www.novartis.us/files/kesimpta.pdf (accessed April 2022).

[2] European Medicines Agency. Kesimpta SmPC, https://www.ema.europa.eu/en/documents/product-information/kesimpta-epar-product-information_en.pdf. (accessed April 2022).

[3] Yu H Graham G David OJ , et al. Population pharmacokinetic-B Cell modeling for ofatumumab in patients with relapsing multiple sclerosis. CNS Drugs 2022; 36(3): 283–300. 35233753 10.1007/s40263-021-00895-w PMC8927028

[4] Hauser SL Bar-Or A Cohen JA. Ofatumumab versus teriflunomide in multiple sclerosis. N Engl J Med 2020; 383: 546–557. 32757523 10.1056/NEJMoa1917246

[5] Novartis Press Release. FDA approves Novartis Kesimpta® (ofatumumab), the first and only self-administered, targeted B-cell therapy for patients with relapsing multiple sclerosis, https://www.novartis.com/news/media-releases/fda-approves-novartis-kesimpta-ofatumumab-first-and-only-self-administered-targeted-b-cell-therapy-patients-relapsing-multiple-sclerosis (accessed March 2023).

[6] Bar-Or A Wiendl H Montalban X , et al. Rapid and sustained B-cell depletion with subcutaneous ofatumumab in relapsing multiple sclerosis: APLIOS, a randomized phase-2 study. Mult Scler 2022; 28(6): 910–924. 34605319 10.1177/13524585211044479 PMC9024029

[7] Kira JI Nakahara J Sazonov DV , et al. Effect of ofatumumab versus placebo in relapsing multiple sclerosis patients from Japan and Russia: Phase 2 APOLITOS study. Mult Scler 2021; 28: 1229–1238. 34787005 10.1177/13524585211055934

[8] Hauser SL Cross AH Winthrop K , et al. Safety experience with continued exposure to ofatumumab in patients with relapsing forms of multiple sclerosis for up to 3.5 years. Mult Scler 2022; 28(10): 1576–1590. 35229668 10.1177/13524585221079731 PMC9330270

[9] Harding K Williams O Willis M , et al. Clinical outcomes of escalation vs early intensive disease-modifying therapy in patients with multiple sclerosis. JAMA Neurol 2019; 76: 536–541. 30776055 10.1001/jamaneurol.2018.4905 PMC6515582

[10] He A Merkel B Brown JWL , et al. Timing of high-efficacy therapy for multiple sclerosis: A retrospective observational cohort study. Lancet Neurol 2020; 19(4): 307–316. 32199096 10.1016/S1474-4422(20)30067-3

[11] Iaffaldano P Lucisano G Caputo F , et al. Long-term disability trajectories in relapsing multiple sclerosis patients treated with early intensive or escalation treatment strategies. Ther Adv Neurol Disord 2021; 14: 17562864211019574. 34104220 10.1177/17562864211019574 PMC8170278

[12] Gärtner J Hauser SL Bar-Or A , et al. Efficacy and safety of ofatumumab in recently diagnosed, treatment-naive patients with multiple sclerosis: Results from ASCLEPIOS I and II. Mult Scler 2022; 28(10): 1562–1575. 35266417 10.1177/13524585221078825 PMC9315184

[13] US Department of Health and Human Services. Common Terminology Criteria for Adverse Events (CTCAE) version 5.0. 2017, https://ctep.cancer.gov/protocoldevelopment/electronic_applications/docs/ctcae_v5_quick_reference_8.5x11.pdf (accessed April 2022).

[14] van Brummelen EM Ros W Wolbink G , et al. Antidrug antibody formation in oncology: Clinical relevance and challenges. Oncologist 2016; 21(10): 1260–1268. 27440064 10.1634/theoncologist.2016-0061 PMC5061540

[15] Hauser SL Bar-Or A Cohen JA , et al. Ofatumumab versus teriflunomide in multiple sclerosis. N Engl J Med 2020; 383: 546–557, https://www.nejm.org/doi/full/10.1056/NEJMoa1917246#article_supplementary_material (accessed July 2023). 32757523 10.1056/NEJMoa1917246

[16] Chaudhry F Jageka C Levy PD , et al. Review of the COVID-19 risk in multiple sclerosis. J Cell Immunol 2021; 3: 68–77. 33959727 10.33696/immunology.3.080 PMC8098748

[17] Druyan A Lidar M Brodavka M , et al. The risk for severe COVID 19 in patients with autoimmune and/or inflammatory diseases: First wave lessons. Dermatol Ther 2021; 34: e14627. 10.1111/dth.14627 PMC7883029 33277764

[18] Wijnands JM Kingwell E Zhu F , et al. Infection-related health care utilization among people with and without multiple sclerosis. Mult Scler 2017; 23(11): 1506–1516. 28273769 10.1177/1352458516681198

[19] Winkelmann A Loebermann M Reisinger EC , et al. Disease-modifying therapies and infectious risks in multiple sclerosis. Nat Rev Neurol 2016; 12(4): 217–233. 26943779 10.1038/nrneurol.2016.21

[20] Louapre C Ibrahim M Maillart E , et al. Anti-CD20 therapies decrease humoral immune response to SARS-CoV-2 in patients with multiple sclerosis or neuromyelitis optica spectrum disorders. J Neurol Neurosurg Psychiatry 2022; 93: 24–31. 34341142 10.1136/jnnp-2021-326904

[21] Gadani SP Reyes-Mantilla M Jank L , et al. Discordant humoral and T cell immune responses to SARS-CoV-2 vaccination in people with multiple sclerosis on anti-CD20 therapy. EbioMedicine 2021; 73: 103636. 34666226 10.1016/j.ebiom.2021.103636 PMC8520057

[22] Novak F Nilsson AC Nielsen C , et al. Humoral immune response following SARS-CoV-2 mRNA vaccination concomitant to anti-CD20 therapy in multiple sclerosis. Mult Scler Relat Disord 2021; 56: 103251. 34571415 10.1016/j.msard.2021.103251 PMC8426319

[23] Sormani MP Inglese M Schiavetti I , et al. Effect of SARS-CoV-2 mRNA vaccination in MS patients treated with disease modifying therapies. EbioMedicine 2021; 72: 103581. 34563483 10.1016/j.ebiom.2021.103581 PMC8456129

[24] Cross AH Delgado S Habek M , et al. COVID-19 outcomes and vaccination in people with relapsing multiple sclerosis treated with ofatumumab. Neurol Ther 2022; 11: 741–758. 35284994 10.1007/s40120-022-00341-z PMC8918079

[25] World Health Organization. WHO coronavirus (COVID-19) dashboard. World Health Organization, https://covid19.who.int/ (accessed February 2023).

[26] Ziemssen T De Stefano N Sormani MP , et al. Optimizing therapy early in multiple sclerosis: An evidence-based view. Mult Scler Relat Disord 2015; 4(5): 460–469. 26346796 10.1016/j.msard.2015.07.007

