# Supplementary material for: Efficacy and safety of four-year ofatumumab treatment in relapsing multiple sclerosis: The ALITHIOS open-label extension
Source: Mult Scler. 2023 Sep 11;29(11-12):1452–64. doi: 10.1177/13524585231195346 (PMC10580679; doi:10.1177/13524585231195346)
Supplement: sj-docx-1-msj-10.1177_13524585231195346 – Supplemental material for Efficacy and safety of four-year ofatumumab treatment in relapsing multiple sclerosis: The ALITHIOS open-label extension [file sj-docx-1-msj-10.1177_13524585231195346.docx]

# SUPPLEMENTARY MATERIAL

# Key protocol amendments

During ASCLEPIOS I/II, APLIOS and APOLITOS, investigators were required to interrupt treatment if immunoglobulin M (IgM) levels fell below 10% LLN, or IgG levels fell below 20% LLN; treatment was not to resume until IgM or IgG levels returned to within normal limits. This requirement was removed by a protocol amendment (June 03, 2021) for ALITHIOS and left to the discretion of the investigator.

# Key inclusion and exclusion criteria for the ALITHIOS open-label extension

Eligibility criteria at screening included: 18–55 years of age (inclusive), a diagnosis of multiple sclerosis (MS; according to the 2010 revised McDonald criteria)^1^ with a relapsing-remitting course or a secondary progressive course with disease activity (according to the Lublin criteria)^2^, expanded Disability Status Scale (EDSS) score of 0–5.5, ≥1 one relapse in the year before screening, or ≥2 relapses in the two years before screening, or ≥1 gadolinium-enhancing (Gd+) lesion detected on MRI in the year before randomization, and neurologically stable for ≥1 month prior to randomization. Key exclusion criteria included: a diagnosis of primary progressive MS or secondary progressive MS without disease activity, neuromyelitis optica, disease duration of >10 years with an EDSS score of ≤2.0, neurological findings consistent with progressive multifocal leukoencephalopathy (PML) or confirmed PML, recipient of live or live attenuated vaccines during the two months prior to randomization, or previous treatment with DMTs.^3^

# Trial oversight

Prior to commencement, study protocols were approved by an institutional review board or ethics committee at each site, and all randomized patients provided written informed consent prior to receiving trial treatment. All data were collected by site investigators and then analyzed by Novartis Pharmaceuticals. The trials were conducted in accordance with the International Conference on Harmonization guidelines for Good Clinical Practice^4^ and the principles of the Declaration of Helsinki^5^, while U.S sites maintained compliance with Health Insurance Portability and Accountability Act regulations.^6^

#

# Endpoints: definitions and assessment criteria

## Annualized relapse rate (ARR)

The ARR per study period (core and extension) was defined as the number of confirmed MS relapses per year. A relapse was defined as the appearance of a new neurological abnormality or worsening of a pre-existing neurological abnormality (persisting for ≥24 hours), separated by ≥30 days from the onset of a preceding clinical demyelinating event. The definition of a confirmed MS relapse was one accompanied by a clinically relevant change in the EDSS performed by the EDSS Rater, i.e., an increase of ≥0.5 points on the EDSS score, or an increase of 1 point on two functional scores (FSs) or 2 points on one FS, excluding changes involving bowel/bladder or cerebral FS compared to the previous available rating (the last EDSS rating that did not occur during a relapse). Confirmation of MS relapse based on these definitions was done centrally.

## Confirmed disability worsening at 3 months (3mCDW) and 6 months (6mCDW)

CDW was defined as an increase from baseline in EDSS score sustained for ≥3 or 6 months, respectively. Therefore, after a scheduled or unscheduled visit at which the patient fulfills the disability worsening criterion, all EDSS assessments (scheduled or unscheduled) need to also fulfill the worsening criteria until the worsening (“the event”) can be confirmed at the first scheduled visit that occurs 3 months (or 6 months) after the onset of the worsening, or later. Censoring occurs in all patients who did not experience a 3mCDW (or 6mCDW) event in the study (censoring also occurs in patients who had a “tentative” disability worsening that could not be confirmed due to an early discontinuation or any another reason). The censoring time is defined as the time from the first dose to the last available EDSS assessment.

## Acute brain MRI lesion activity

The number of Gd+T1 lesions was assessed every 48 weeks. The number of new or enlarging T2 lesions was assessed every year.

## Serum neurofilament light chain (sNFL) concentration

Neurofilament light chain is a specific biomarker of neuro-axonal injury, released into the cerebrospinal fluid and serum following neuro-axonal damage. Serum and cerebrospinal fluid concentrations are highly correlated, and sNfL concentration is higher in those with MS versus age-matched, healthy controls.^7^ Two assays were used to assess sNfL: for ASCLEPIOS I/II (core period) the Quanterix Simoa^®^ NF-light™ Advantage Kit validated at Navigate BioPharma Services (Carlsbad, CA, USA) assay was used; for ALITHIOS (extension period) the Siemens Healthineers NfL laboratory developed a test on the Atellica^®^ Immunoassay system that was validated at Siemens Healthcare Laboratory (Berkeley, CA, USA). A good correlation between the two assays was observed (Pearson’s correlation = 0.995; average quantitation difference of 8%). To assess the long-term treatment effect on sNfL in the overall period, it was necessary to pool the core and extension data. As the two assays were not equivalent, a transformation algorithm (from the Quanterix Simoa assay to the Siemens Atellica assay) was established by Siemens. These “assay-transformed values” can be calculated as 2.06 + 0.83*original values.

## NEDA-3 status

NEDA-3 was defined as no 6mCDW events, no confirmed relapses, and no MRI activity (new Gd+ T1 lesions, or neT2 lesions).

## Interruption and discontinuation requirements related to IgG and IgM

In the core studies, investigators were required to interrupt treatment if IgM levels fell below 10% LLN, or IgG below 20% LLN, and treatment was not to be resumed until IgM/IgG level returned to within normal limits; however, in the extension study the requirement to interrupt treatment was removed by protocol amendment two and thereafter left to the discretion of the investigators. Sensitivity analyses were conducted to determine whether early interruption/discontinuation of ofatumumab due to low IgG/IgM impacted overall Ig trends, or the stabilization of IgG. Imputation of missing IgG and IgM data was performed for IgG by last observation carried forward (LOCF) and for IgM, by LOCF and half LLN (Supplementary Figures 5E, 5F and 5G).

# Injection-related reactions (IRRs): additional information

IRRs are common with high-dose intravenous anti-CD20 therapies, with premedication often required to reduce their frequency and severity.^8, 9^ Ofatumumab uses s.c. administration. and in the current analysis almost all systemic and local-site IRRs with the first injection were mild to moderate, and non-serious (both in >99% patients).

**References**

1. Polman CH, Reingold SC, Banwell B, et al. Diagnostic criteria for multiple sclerosis: 2010 revisions to the McDonald criteria. *Ann Neurol* 2011; 69: 292-302. 2011/03/10. DOI: 10.1002/ana.22366.

2. Lublin FD, Reingold SC, Cohen JA, et al. Defining the clinical course of multiple sclerosis: The 2013 revisions. *Neurology* 2014; 83: 278–286. 2014/05/30. DOI: 10.1212/WNL.0000000000000560.

3. Hauser SL, Bar-Or A and Cohen JA. Ofatumumab versus Teriflunomide in Multiple Sclerosis. *N Engl J Med* 2020; 383: 546-557. DOI: DOI: 10.1056/NEJMoa1917246.

4. International Conference on Harmonisation of technical requirements for registration of pharmaceuticals for human use. ICH harmonized tripartite guideline: Guideline for Good Clinical Practice. *J Postgrad Med* 2001; 47: 45-50.

5. World Medical Association. WMA Declaration of Helsinki - ethical principles for medical research involving human subjects., <https://www.wma.net/policies-post/wma-declaration-of-helsinki-ethical-principles-for-medical-research-involving-human-subjects/> (2018, accessed April 2022).

6. Centers for Disease Control and Prevention (CDC). Health Insurance Portability and Accountability Act of 1996 (HIPAA). Avilable at: <https://www.cdc.gov/phlp/publications/topic/hipaa.html> (acccessed March 2022).

7. Disanto G, Barro C, Benkert P, et al. Serum Neurofilament light: A biomarker of neuronal damage in multiple sclerosis. *Ann Neurol* 2017; 81: 857-870. 2017/05/18. DOI: 10.1002/ana.24954.

8. European Medicines Agency. Kesimpta SmPC <https://www.ema.europa.eu/en/documents/product-information/kesimpta-epar-product-information_en.pdf>. (accessed April 2022)

9. US Food and Drug Administration. KESIMPTA^®^ (ofatumumab) Prescribing Information. <https://www.novartis.us/sites/www.novartis.us/files/kesimpta.pdf> (2020, accessed April 2022).

# Supplementary tables

## Supplementary Table 1. Patient disposition: patients who entered ALITHIOS (efficacy analysis set)

|  | **Entered ALITHIOS (*N*=1367)** | |
| --- | --- | --- |
| **Disposition/Reason for discontinuation*** | **Ofatumumab *N*=690 *n* (%)** | **Teriflunomide**  ***N*=677 *n* (%)** |
| Ongoing at data cut-off | 613 (88.8) | 601 (88.8) |
| Discontinued during open-label extension period | 77 (11.2) | 76 (11.2) |
| Primary reason of discontinuation |  |  |
| Adverse event | 29 (4.2) | 26 (3.8) |
| Subject/guardian decision | 25 (3.6) | 29 (4.3) |
| Lost to follow-up | 6 (0.9) | 1 (0.1) |
| Physician decision | 5 (0.7) | 5 (0.7) |
| Lack of efficacy | 4 (0.6) | 6 (0.9) |
| Death | 3 (0.4) | 3 (0.4) |
| Pregnancy | 3 (0.4) | 4 (0.6) |
| Non-compliance with study treatment | 1 (0.1) | 1 (0.1) |
| Protocol deviation | 1 (0.1) | 1 (0.1) |

*Data cut-off: 25-Sep-2021. Data from the efficacy analysis set. Data report patient disposition of randomized patients in ASCLEPIOS I/II who entered ALITHIOS.

***Supplementary Table 2. Baseline demographics and clinical characteristics (safety analysis set)***

|  | **Continuous ofatumumab^a^ (*N*=1292)** | **Newly-switched ofatumumab group**  **(*N*=677)** | | **Overall ofatumumab (*N*=1969)** |
| --- | --- | --- | --- | --- |
|  |  | **Baseline of core period** | **Baseline of open-label extension period** |  |
| Age, years (mean±SD) | 38.0±9.06 | 38.2±9.22 | 40.1±9.21 | 38.7±9.16 |
| BMI, kg/m^2^ (mean±SD) | 25.61±6.16 | 25.69±5.83 | 25.61±5.85 | 25.61±6.05 |
| Female, n (%) | 889 (68.8) | 456 (67.4) | 456 (67.4) | 1345 (68.3) |
| Time since MS symptom onset, years (mean±SD) | 8.48±7.33 | 8.06±7.21 | 9.94±7.23 | 8.98±7.33 |
| Time since diagnosis, years (mean±SD) | 5.87±6.31 | 5.45±6.00 | 7.33±6.01 | 6.37±6.25 |
| Type of MS at study entry, n (%) |  |  |  |  |
| RRMS | 1223 (94.7) | 646 (95.4) |  | 1869 (94.9) |
| SPMS | 69 (5.3) | 31 (4.6) |  | 100 (5.1) |
| EDSS score at baseline, (mean±SD) | 2.90±1.33 | 2.77±1.32 | 2.81±1.46 | 2.87±1.38 |
| IgG levels at baseline, g/L (mean±SD) | 10.31±2.24 | 10.35±2.09 | 10.23±2.14 | 10.28±2.21 |
| IgM levels at baseline, g/L (mean±SD) | 1.34±0.65 | 1.36±0.74 | 1.14±0.67 | 1.2 ±0.66 |
|  |  |  |  |  |
| Time-at-risk, median duration; months | 35.8 | 26.0 | 26.0 | 28.1 |
| Time-at-risk, total; PYs | 3831.6 | 1366.2 | 1366.2 | 5197.9 |

Data from the safety analysis set. ^a^Patients who received ≥1 dose of ofatumumab in ASCLEPIOS I/II, APLIOS or APOLITOS.

BMI: body mass index; EDSS: Expanded Disability Status Scale; Ig: immunoglobulin; MS: multiple sclerosis; PYs: patient years; SD: standard deviation. For newly-switched ofatumumab patients, the baseline values from the extension study contributed to the overall summary.

## Supplementary Table 3. Duration of exposure to study treatment in ASCLEPIOS I/II (safety analysis set)

|  | **Continuous ofatumumab group** | | | **Newly-switched ofatumumab group** | | |
| --- | --- | --- | --- | --- | --- | --- |
|  | **Ofatumumab in core period  (*N*=946)** | **Ofatumumab in open-label extension period (*N*=690)** | **Overall  ofatumumab (*N*=946)** | **Teriflunomide in core period (*N*=936)** | **Ofatumumab in open-label extension period  (*N*=677)** | **Overall  teriflunomide + ofatumumab (*N*=936)** |
| Patient-years | 1486.7 | 1277.0 | 2761.4 | 1397.8 | 1271.1 | 2668.9 |
| Duration of exposure, years |  |  |  |  |  |  |
| Mean | 1.6 | 1.9 | 2.9 | 1.5 | 1.9 | 2.9 |
| Q1 | 1.4 | 1.8 | 1.7 | 1.4 | 1.8 | 1.7 |
| Median | 1.6 | 2.1 | 3.5 | 1.6 | 2.1 | 3.4 |
| Q3 | 1.9 | 2.2 | 3.9 | 1.8 | 2.2 | 3.8 |

Data from the safety analysis set. Q1: first/lower quartile; Q3: third/upper quartile.

## Supplementary Table 4. Adherence to study treatment in ASCLEPIOS I/II (safety analysis set)

| **Compliance to study treatment** | **Ofatumumab 20 mg** | | **Teriflunomide 14mg** |  |
| --- | --- | --- | --- | --- |
|  | **Continuous ofatumumab group (*N*=946)** | **Newly-switched ofatumumab group (*N*=677)** | **Newly-switched ofatumumab group (*N*=936)** | |
| Summary | | | | |
| Mean, % | 96.56 | 95.41 | 98.74 | |
| Q1, % | 96.65 | 96.32 | 99.42 | |
| Median, % | 99.42 | 99.52 | 100.00 | |
| Q3, % | 100.00 | 100.00 | 100.00 | |
| Compliance categories, n (%) | | | | |
| =100% | 281 (29.7) | 265 (39.1) | 579 (61.9) | |
| ≥98% | 634 (67.0) | 441 (65.1) | 825 (88.1) | |
| ≥95% | 764 (80.8) | 538 (79.5) | 879 (93.9) | |
| ≥90% | 847 (89.5) | 586 (86.6) | 907 (96.9) | |
| ≥80% | 905 (95.7) | 639 (94.4) | 922 (98.5) | |
| ≥70% | 936 (98.9) | 652 (96.3) | 930 (99.4) | |
| ≥60% | 942 (99.6) | 661 (97.6) | 933 (99.7) | |

Data from the safety analysis set. %-adherence was calculated as duration of exposure (days)/duration of time on-treatment (days) x 100. Premature discontinuation from study drug was not considered to be non-compliance. Q1: first/lower quartile; Q3: third/upper quartile.

## Supplementary Table 5. Incidence rates of malignancy over time (safety analysis set)

| **Malignancies (preferred terms)** | **Overall ofatumumab**  **(N=1969) *n* (EAIR), [95% CI]** |
| --- | --- |
| All malignancies | 17 (0.33), [0.20–0.53] |
| Basal cell carcinoma | 4 (0.08), [0.03–0.21] |
| Invasive breast carcinoma | 2 (0.04), [0.01–0.15] |
| Breast cancer | 1 (0.02), [0.00–0.14] |
| Intestinal metastasis | 1 (0.02), [0.00–0.14] |
| Invasive ductal breast carcinoma | 1 (0.02), [0.00–0.14] |
| Invasive lobular breast carcinoma | 1 (0.02), [0.00–0.14] |
| Malignant melanoma in situ | 1 (0.02), [0.00–0.14] |
| Non-Hodgkin’s lymphoma recurrent | 1 (0.02), [0.00–0.14] |
| Esophageal squamous cell carcinoma | 1 (0.02), [0.00–0.14] |
| Ovarian cancer | 1 (0.02), [0.00–0.14] |
| Papillary renal cell carcinoma | 1 (0.02), [0.00–0.14] |
| Renal cell carcinoma | 1 (0.02), [0.00–0.14] |
| Triple negative breast cancer | 1 (0.02), [0.00–0.14] |

Data from the safety analysis set. Preferred terms are according to MedDRA version 24.1.

CI: confidence interval; CIF: cumulative incidence function; EAIR: exposure adjusted incidence rate per 100 patient years; PY patient years.

## Supplementary Table 6: Summary of IgG-related AEs leading to early treatment interruption or discontinuation (safety analysis set)

|  | **Continuous ofatumumab** | **Newly-switched ofatumumab** | **Overall ofatumumab^a^** |
| --- | --- | --- | --- |
|  | ***N*=1292  *n* (%)** | ***N*=677  *n* (%)** | ***N*=1969  *n* (%)** |
| Number of patients ≥1 episode | 23 (1.8) | 8 (1.2) | 31 (1.6) |
| Number of patients ≥2 consecutive episodes | 14 (1.1) | 3 (0.4) | 17 (0.9) |
| Number of patients with treatment interruptions^b^ | 2 (0.2) | 0 (0.0) | 2 (0.1) |
| Blood immunoglobulin G decreased | 2 (0.2) | 0 (0.0) | 2 (0.1) |
| Number of patients with treatment discontinuations^b^ | 4 (0.3) | 0 (0.0) | 4 (0.2) |
| Immunoglobulins decreased | 2 (0.2) | 0 (0.0) | 2 (0.1) |
| Blood immunoglobulin G abnormal | 1 (0.1) | 0 (0.0) | 1 (0.1) |
| Blood immunoglobulin G decreased | 1 (0.1) | 0 (0.0) | 1 (0.1) |
| Number of patients with ≥1 infection AE | 789 (61.1) | 351 (51.8) | 1140 (57.9) |
| Number of patients with concomitant treatment received for infections | 673 (52.1) | 294 (43.4) | 967 (49.1) |
| Number of patients with infection AEs resolved | 780 (60.4) | 343 (50.7) | 1123 (57.0) |
| Number of patients with ≥1 infection AE by Grade | | | |
| Grade 1 | 271 (21.0) | 121 (17.9) | 392 (19.9) |
| Grade 2 | 466 (36.1) | 215 (31.8) | 681 (34.6) |
| Grade 3 | 48 (3.7) | 13 (1.9) | 61 (3.1) |
| Grade 4 | 4 (0.3) | 2 (0.3) | 6 (0.3) |
| Number of patients with ≥1 infection AE by action taken | | | |
| Dose not changed^c^ | 719 (55.7) | 326 (48.2) | 1045 (53.1) |
| Drug interrupted | 53 (4.1) | 23 (3.4) | 76 (3.9) |
| Drug withdrawn | 5 (0.4) | 1 (0.1) | 6 (0.3) |

Data from the safety analysis set.

^a^Patients who received ≥1 dose of ofatumumab in ASCLEPIOS I/II, APLIOS or APOLITOS. ^b^Interruptions and discontinuations related to preferred terms: Blood immunoglobulin G decreased, Blood Immunoglobulin G abnormal, Immunoglobulins decreased, Selective IgG immunodeficiency, post first IgG decline for patients with ≥1 episode. ^c^No patients had a dose increase or reduction as an action following an infection AE.

AE: adverse event; IgG: immunoglobulin G; NA: not applicable.

## Supplementary Table 7: Summary of IgM-related AEs leading to early treatment interruption or discontinuation (safety analysis set)

|  | **Continuous ofatumumab** | **Newly-switched ofatumumab** | **Overall ofatumumab^a^** |
| --- | --- | --- | --- |
|  | ***N*=1292**  ***n* (%)** | ***N*=677  *n* (%)** | ***N*=1969  *n* (%)** |
| Number of patients ≥1 episode | 357 (27.6) | 166 (24.5) | 523 (26.6) |
| Number of patients ≥2 consecutive episodes | 278 (21.5) | 115 (17.0) | 393 (20.0) |
| Number of patients with treatment interruptions^b^ | 135 (10.4) | 58 (8.6) | 193 (9.8) |
| Blood immunoglobulin M decreased | 129 (10.0) | 56 (8.3) | 185 (9.4) |
| Immunoglobulins decreased | 9 (0.7) | 1 (0.1) | 10 (0.5) |
| Hypogammaglobulinemia | 0 (0.0) | 1 (0.1) | 1 (0.1) |
| Hypoglobulinemia | 0 (0.0) | 1 (0.1) | 1 (0.1) |
| Number of patients with treatment discontinuations^b^ | 57 (4.4) | 14 (2.1) | 71 (3.6) |
| Blood immunoglobulin M decreased | 47 (3.6) | 13 (1.9) | 60 (3.0) |
| Immunoglobulins decreased | 7 (0.5) | 0 (0.0) | 7 (0.4) |
| Hypogammaglobulinemia | 2 (0.2) | 0 (0.0) | 2 (0.1) |
| Hypoglobulinemia | 1 (0.1) | 1 (0.1) | 2 (0.1) |
| Number of patients with ≥1 infection AE | 702 (54.3) | 305 (45.1) | 1007 (51.1) |
| Number of patients with concomitant treatment received for infections | 595 (46.1) | 258 (38.1) | 853 (43.3) |
| Number of patients with infection AEs resolved | 692 (53.6) | 294 (43.4) | 986 (50.1) |

| Number of patients with ≥1 infection AE by Grade | | | |
| --- | --- | --- | --- |
| Grade 1 | 245 (19.0) | 100 (14.8) | 345 (17.5) |
| Grade 2 | 416 (32.2) | 190 (28.1) | 606 (30.8) |
| Grade 3 | 38 (2.9) | 14 (2.1) | 52 (2.6) |
| Grade 4 | 3 (0.2) | 1 (0.1) | 4 (0.2) |
| Number of patients with ≥1 infection AE by action taken | | | |
| Dose not changed^†^ | 642 (49.7) | 283 (41.8) | 925 (47.0) |
| Drug interrupted | 39 (3.0) | 20 (3.0) | 59 (3.0) |
| Drug withdrawn | 5 (0.4) | 1 (0.1) | 6 (0.3) |

Data from the safety analysis set.

^a^Patients who received ≥1 dose of ofatumumab in ASCLEPIOS I/II, APLIOS or APOLITOS. ^b^Interruptions and discontinuations related to preferred terms: Blood immunoglobulin M decreased, Blood Immunoglobulin M abnormal, Immunoglobulins decreased, Selective IgM immunodeficiency, Hypogammaglobulinemia and Hypoglobulinemia post first IgM decline for patients with ≥1 episode. ^†^No patients had a dose increase or reduction as an action following an infection AE.

AE: adverse event; IgM: immunoglobulin M; NA: not applicable.

***Supplementary Table 8. Patients with ≥1 serious infection within 1 month prior and until 1 month after any series of drops in IgG/IgM <LLN (safety analysis set)***

|  | **IgM** | | | | **IgG** | | | | **Overall** | | |
| --- | --- | --- | --- | --- | --- | --- | --- | --- | --- | --- | --- |
|  | **<LLN**  **(*N*=523^†^)** | | **≥LLN**  **(*N*=1443^‡^)** | | **<LLN**  **(*N*=31^†^)** | | **≥LLN**  **(*N*=1935^‡^)** | | ***N*=1969** | | |
|  | ***n* (%)** | **EAIR^§^** | ***n* (%)** | **EAIR^§^** | ***n* (%)** | **EAIR^§^** | ***n* (%)** | **EAIR^§^** | ***n* (%)** | **EAIR^§^** |  |
| Patients with ≥1 serious infection | 6 (1.15) | 1.32 | 55 (3.8) | 1.45 | 1 (3.23) | 6.29 | 75 (3.9) | 1.49 | 78 (3.96) | 1.53 |  |
| Herpes zoster (PT) | 1 (0.2) | 0.22 | 0 | 0 | 0 | 0 | 1 (0.05) | 0.02 | 1 (0.05) | 0.02 |  |
| URTI (PT) | 1 (0.2) | 0.22 | 0 | 0 | 0 | 0 | 1 (0.05) | 0.02 | 1 (0.05) | 0.02 |  |
| UTI (PT) | 2 (0.4) | 0.44 | 3 (0.21) | 0.08 | 0 | 0 | 6 (0.31) | 0.12 | 6 (0.31) | 0.12 |  |
| Bronchitis | 1 (0.2) | 0.22 | 0 | 0 | 0 | 0 | 1 (0.05) | 0.02 | 1 (0.05) | 0.02 |  |
| Pneumonia | 0 | 0 | 8 (0.55) | 0.21 | 1 (3.23) | 6.29 | 8 (0.41) | 0.16 | 9 (0.46) | 0.17 |  |
| COVID-19 | 1 (0.2) | 0.22 | 11 (0.76) | 0.29 | 0 | 0 | 13 (0.7) | 0.25 | 13 (0.66) | 0.25 |  |

Data from the safety analysis set. Preferred terms are according to MedDRA version 24.1.

^†^Number of patients with IgM/IgG <LLN at least once at any time during the post-baseline visits. ^‡^Number of patients with no occurrence of IgM/IgG <LLN at least once at any time during the post-baseline visit. ^§^EAIR per 100 PYs estimated via a Poisson regression model with only treatment as the factor and with the log-link and natural logarithm of time as the offset variable.

Ig: immunoglobulin; EAIR: exposure adjusted incidence rate per 100 patient years; LLN: lower limit of normal; PT: preferred term; PY: patient year.

# Supplementary figures

## Supplementary Figure 1. Between-group comparisons during the core period and extension period (continuous ofatumumab versus switch group) for: (A) ARR^a^; (B) Mean number of Gd-enhancing T1 lesions^b^; (C) Annualized rate of new or enlarging T2 lesions^c^ (efficacy analysis set)

**(A)**


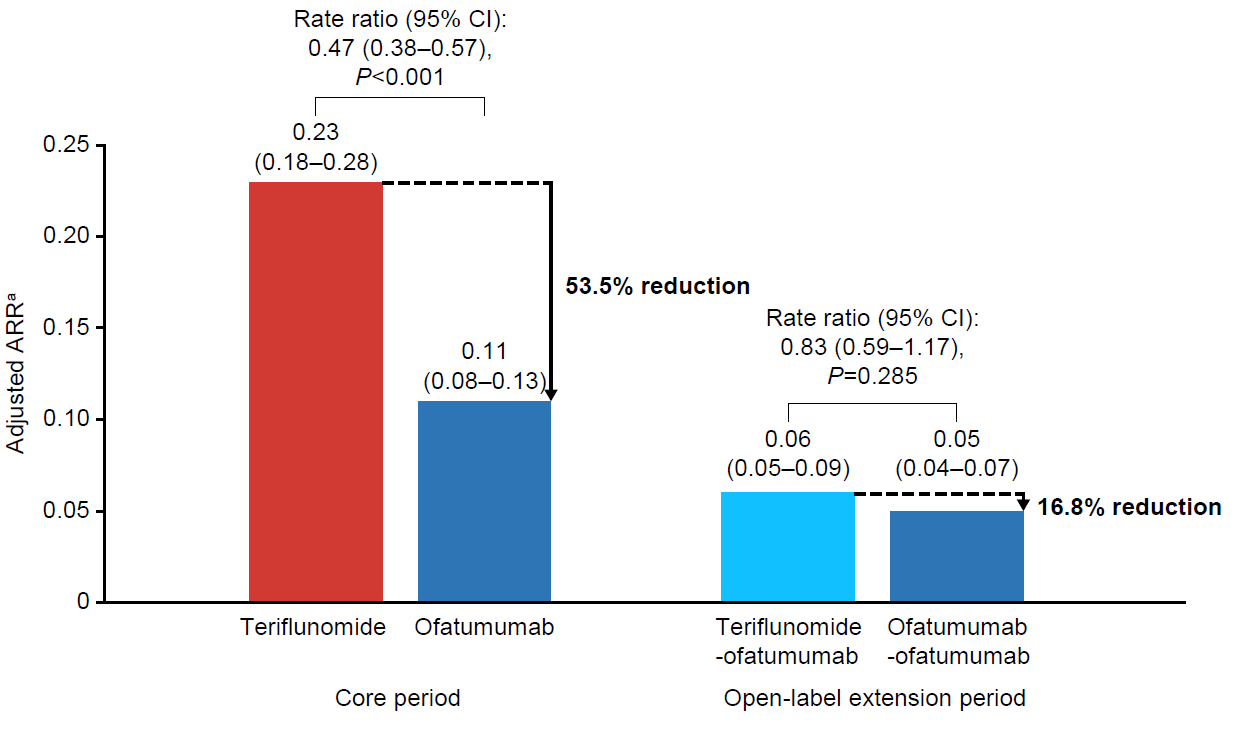


**(B)**


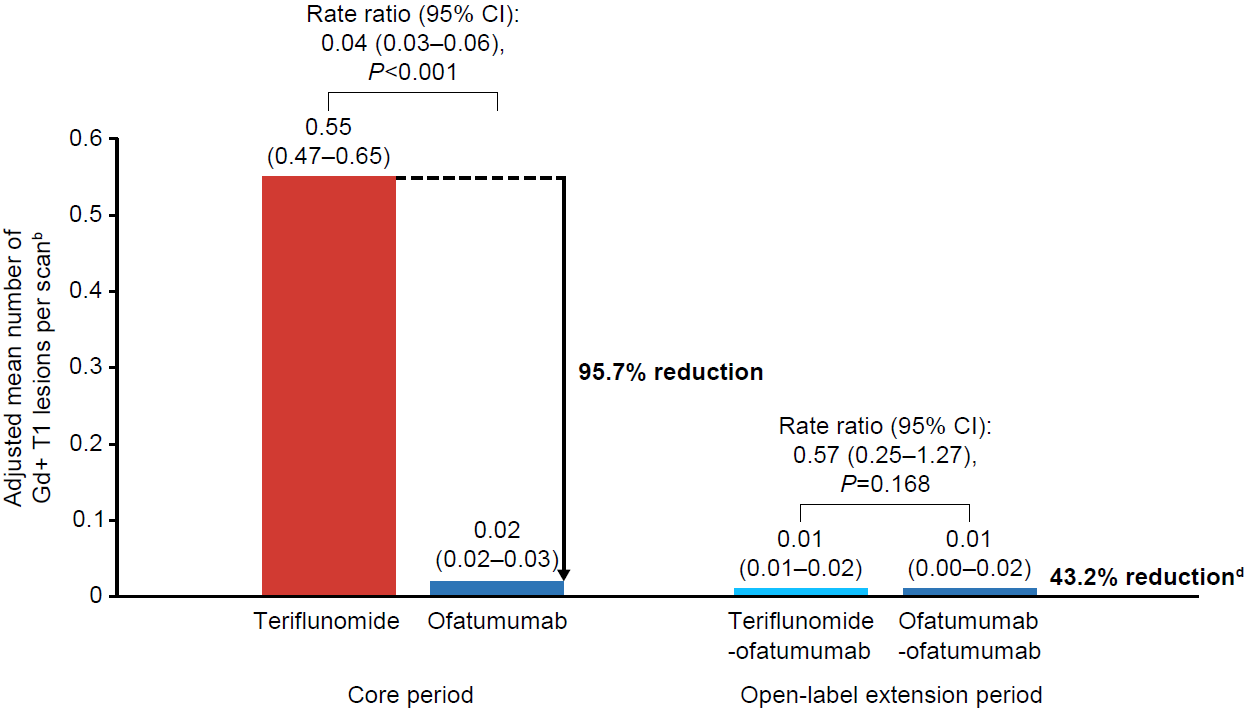


**(C)**


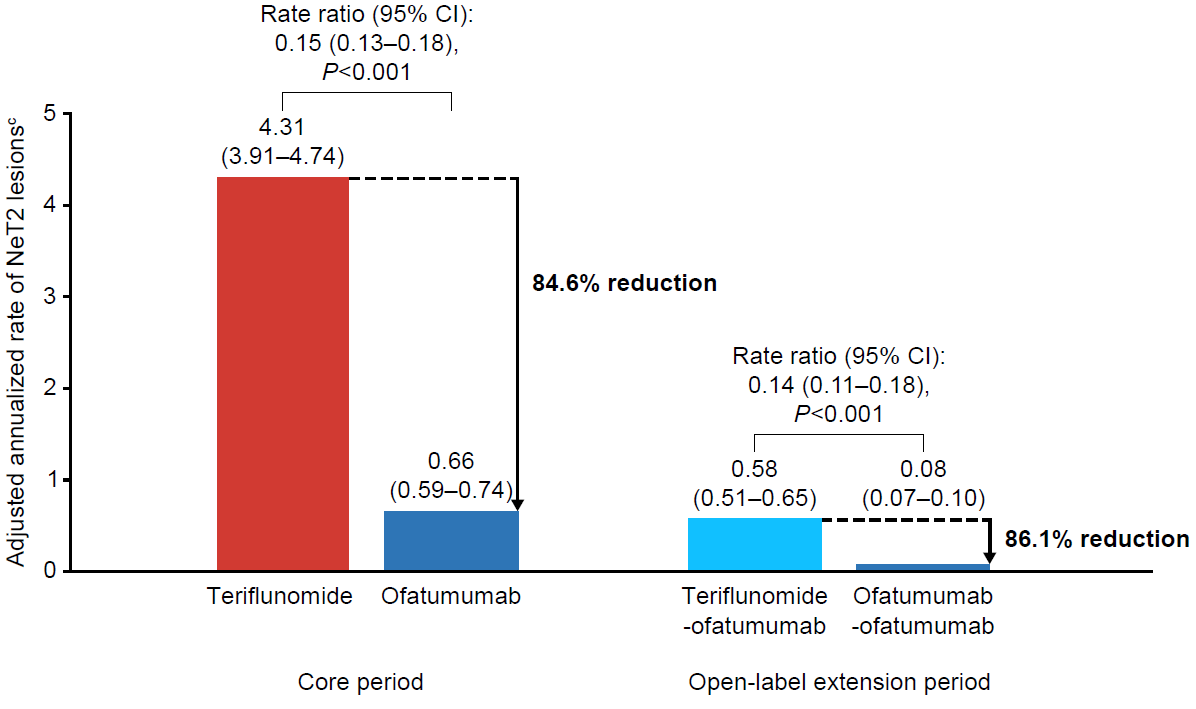


Data from the efficacy analysis set.

^a^Obtained from fitting a piecewise negative binomial model for the time period core period and extension period with log-link, adjusted for treatment and region as factors, number of relapses in previous year, baseline EDSS, baseline number of Gd+ lesions and the patient’s age at baseline as covariates. ^b^Estimated from fitting a piecewise negative binomial model for the core period and extension period with log-link, adjusted for treatment and region as factors, baseline number of T1 Gd+ lesions and patient’s age at baseline as covariates. ^c^Estimated from fitting a piecewise negative binomial model for the core period and extension period with log-link, adjusted for treatment as factor, baseline volume of T2 lesions and patient’s age at baseline as covariates. ^d^%-reduction in annualized rate of neT2 lesions (i.e. the rate in the continuous ofatumumab as a proportion of the rate in the newly switched ofatumumab group) is based on data presented to two decimal places.

ARR: annualized relapse rate, CI: confidence interval; Gd+: gadolinium-enhancing; EDSS: Expanded Disability Status Scale; NeT2 lesions: new/enlarging T2 lesions.

## Supplementary Figure 2: Serum neurofilament light chain (sNfL) concentration: (A) In the core period; (B) In the open-label extension period; (C) In the overall period (cumulative data ≤4 years of treatment) (efficacy analysis set)

**(A)**

**
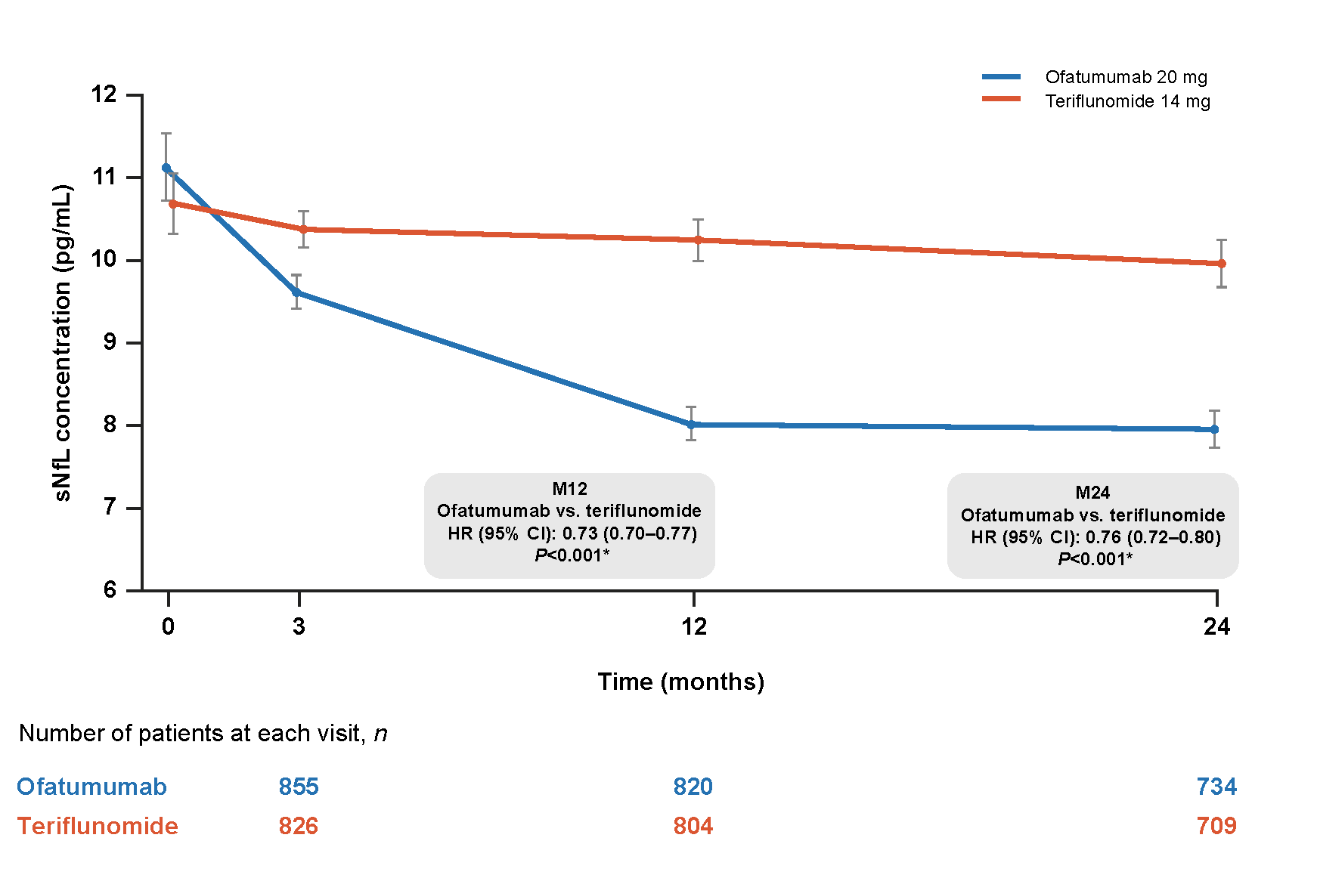
**

**(B)**


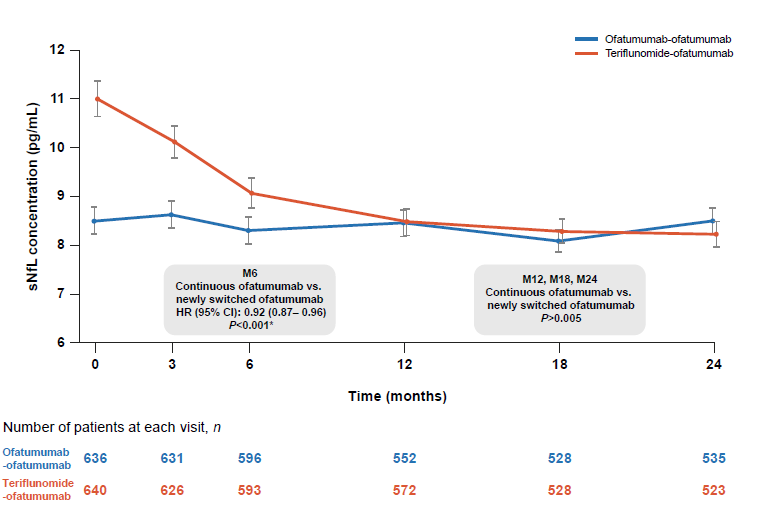


**(C)**

**
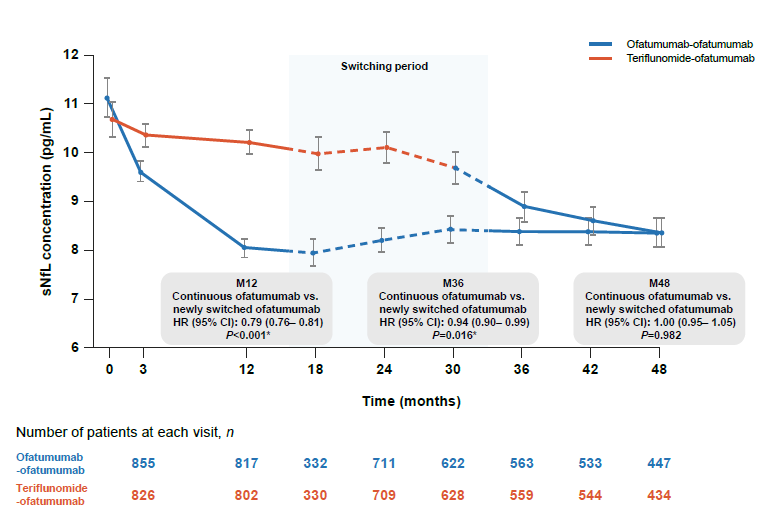
**

Based on the efficacy analysis set. *Statistical significance (two-sided) at the 0.05 level. Data are assay-transformed values (for details of transformation, see “Serum neurofilament light chain (sNfL) assay transformation: rational and methods” in the supplementary materials). Adjusted geometric means with 95% CIs at each time point are from a repeated measures model. Geometric mean sNfL concentration at baseline is derived as the exponentiated arithmetic mean of natural logarithmic of raw values of NfL concentrations.

HR: hazard ratio (adjusted geometric mean ratio); M: month; sNfL: serum neurofilament light chain.

## Supplementary Figure 3. Effect of ofatumumab on individual NEDA-3 components: (A) Patients free of Gd+ T1 lesions; (B) Patients free of new/enlarging T2 lesions; (C) Patients free of confirmed relapses; (D) Patients free of 6mCDW (modified efficacy analysis set)

**(A)**

**
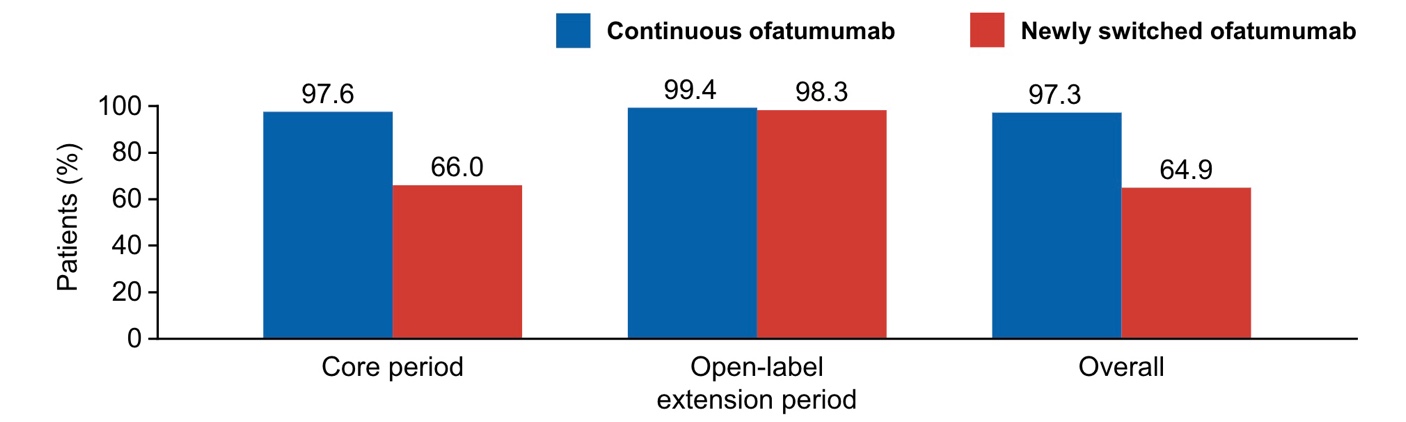
**

**(B)**

**(C)
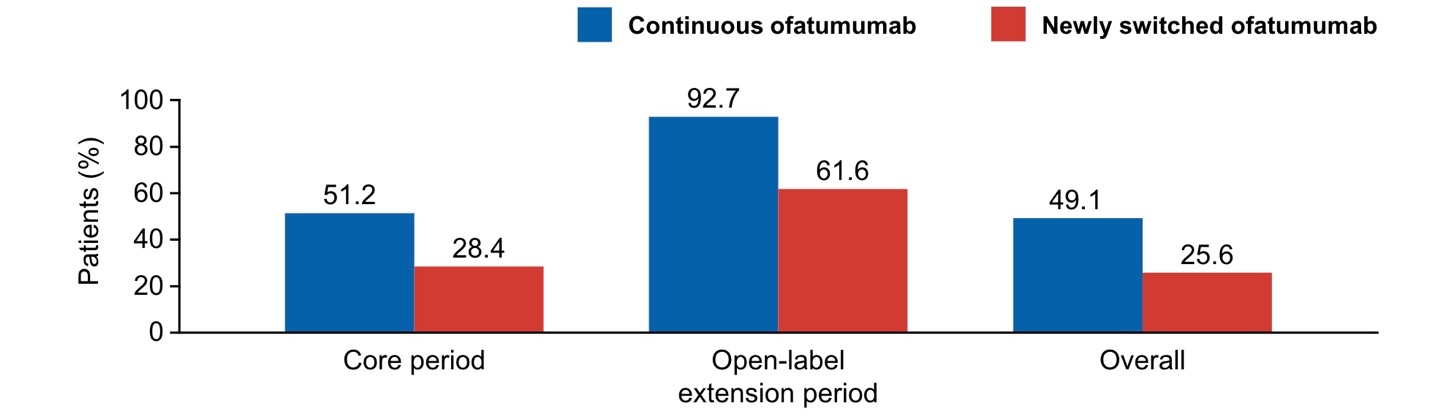
**

**(D)**
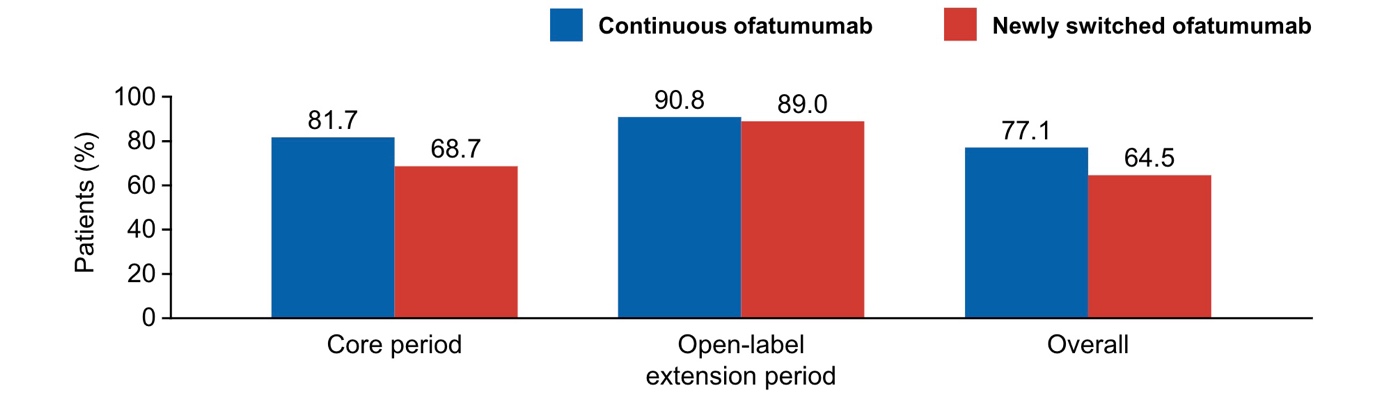


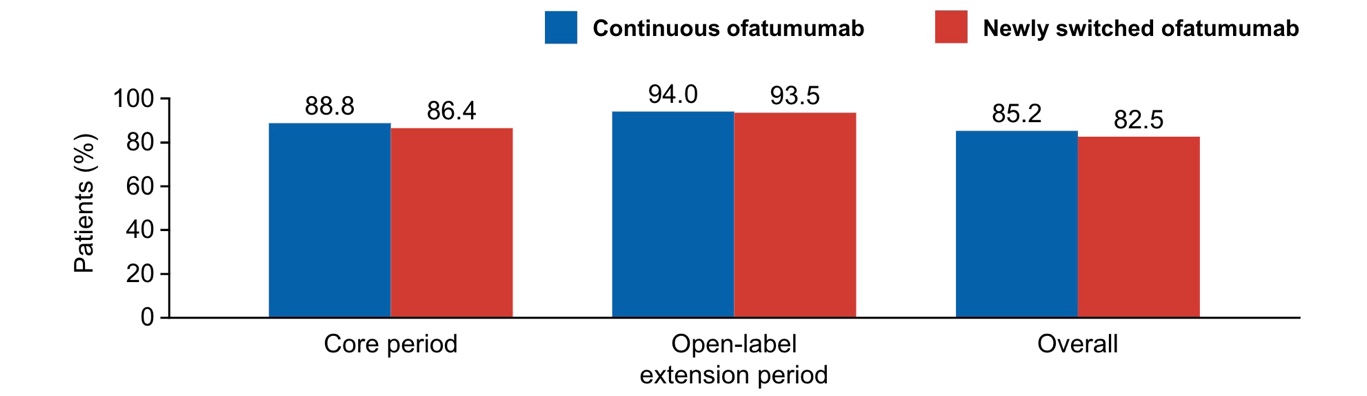


Data from the modified efficacy analysis set for NEDA-3.

CDW: confirmed disability worsening; NEDA-3: three parameter no evidence of disease activity.

## Supplementary Figure 4. Incidence of: (A) Systemic reactions; (B) Injection-site related reactions (safety analysis set)

**(A)**
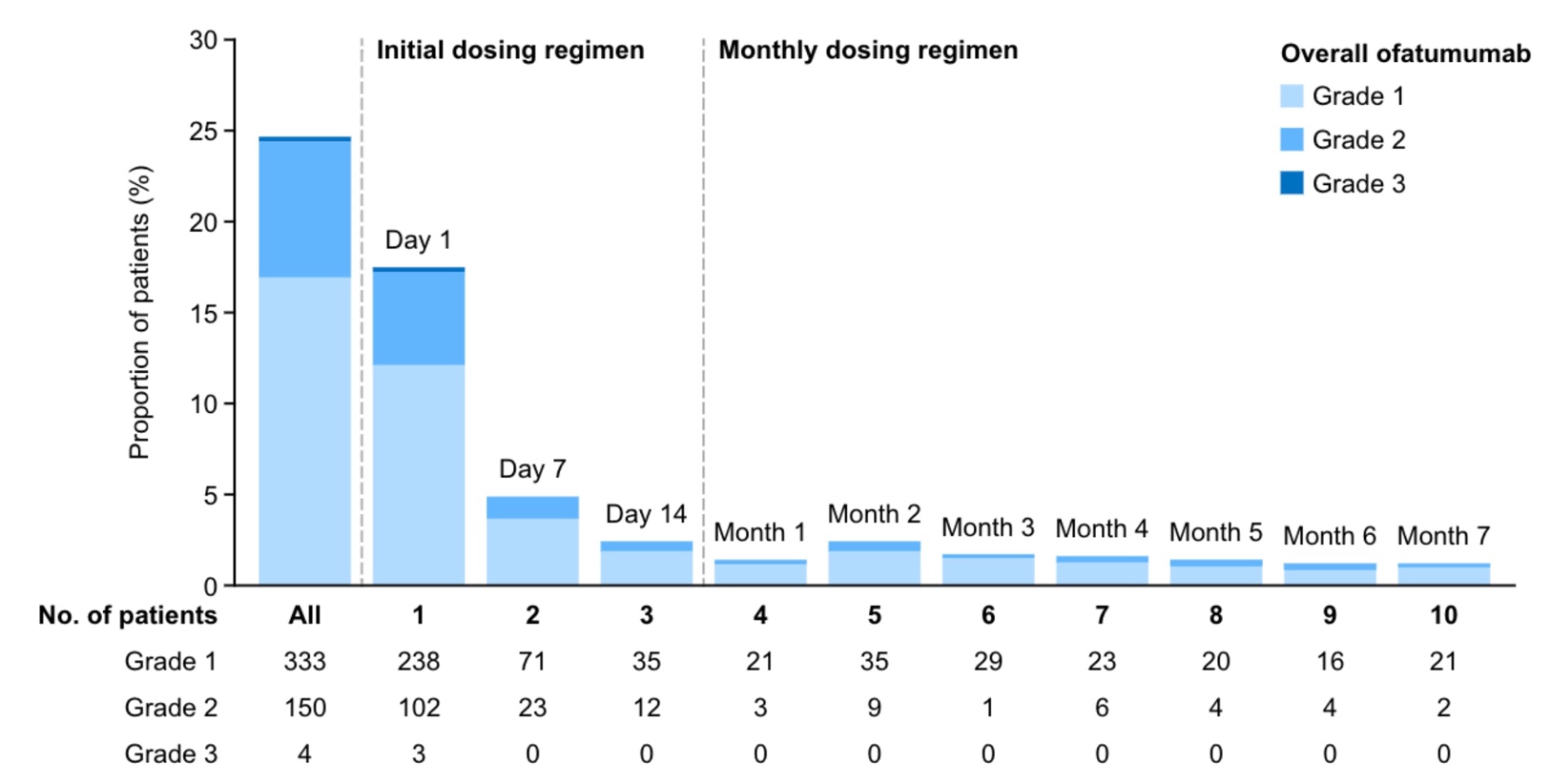


**(B)**

**
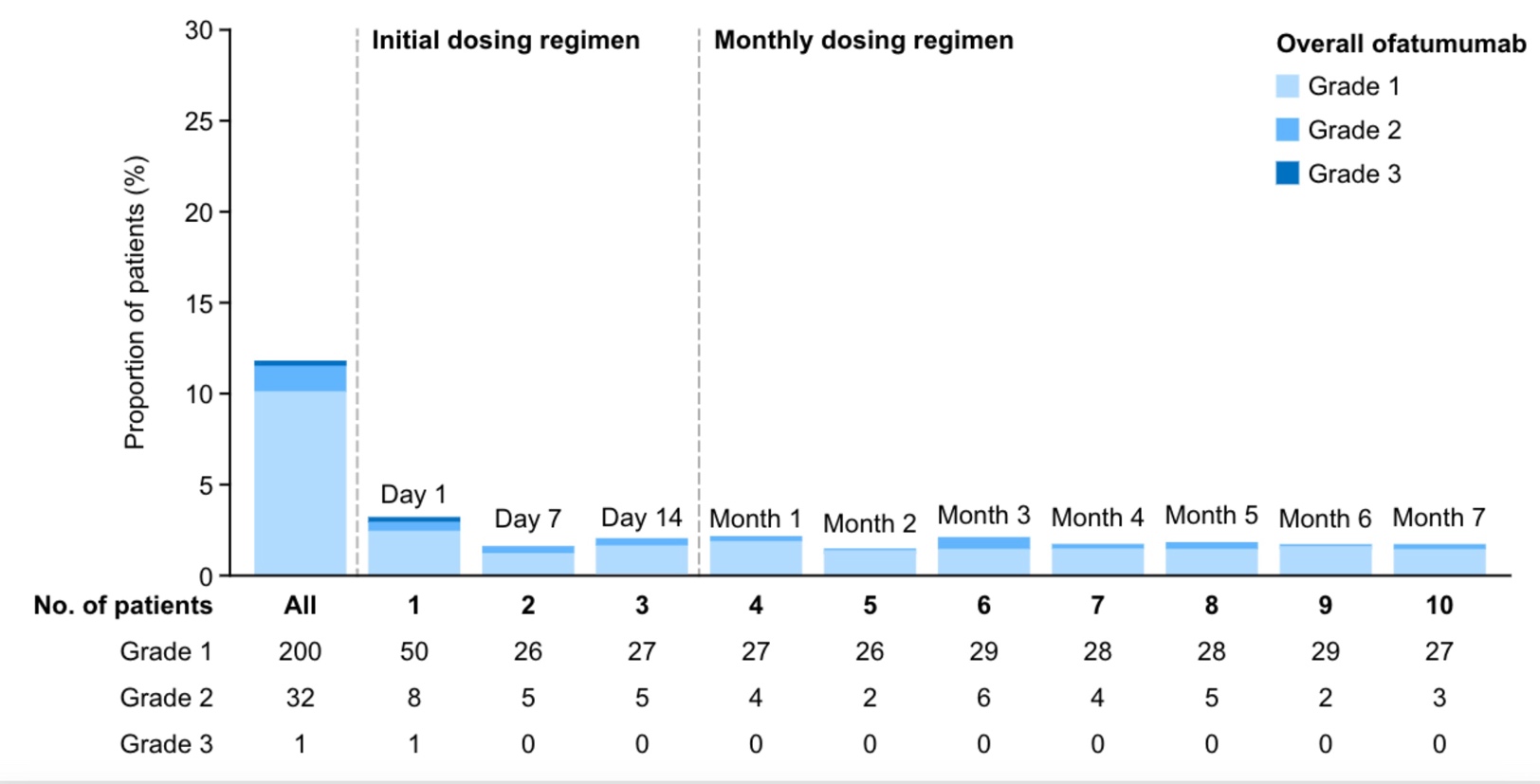
**

Data from the safety analysis set.

## Supplementary Figure 5. Serum Ig levels following up to 4 years of treatment: (A) IgG; (B) IgM; (C); Effect of baseline IgG on IgG levels from first dose of ofatumumab*; (D) Effect of baseline IgM on IgM levels from the first dose of ofatumumab*; (E) IgG sensitivity analysis (± LOCF imputation); (F) IgM sensitivity analysis (± LOCF imputation); (G); IgM sensitivity analysis (± imputation by ½ LLN) (safety analysis set)

**(A)**


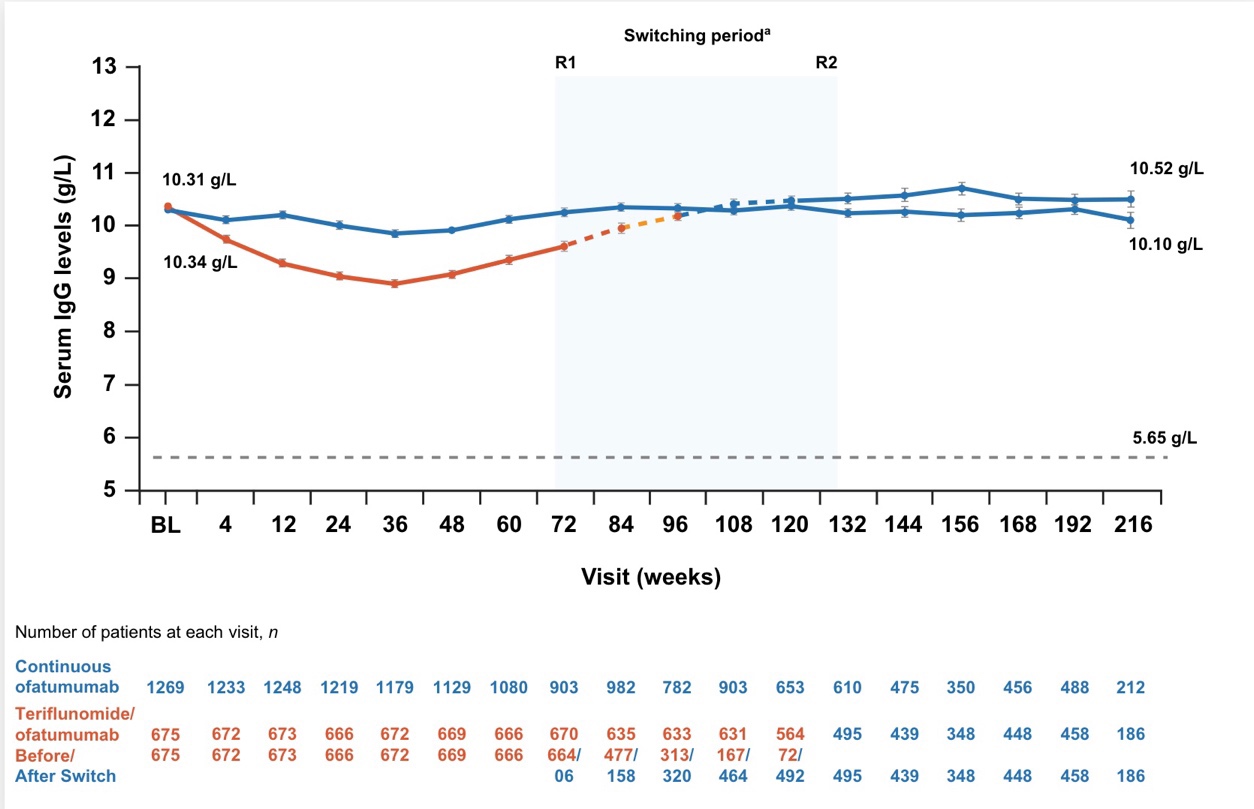


**(B)**


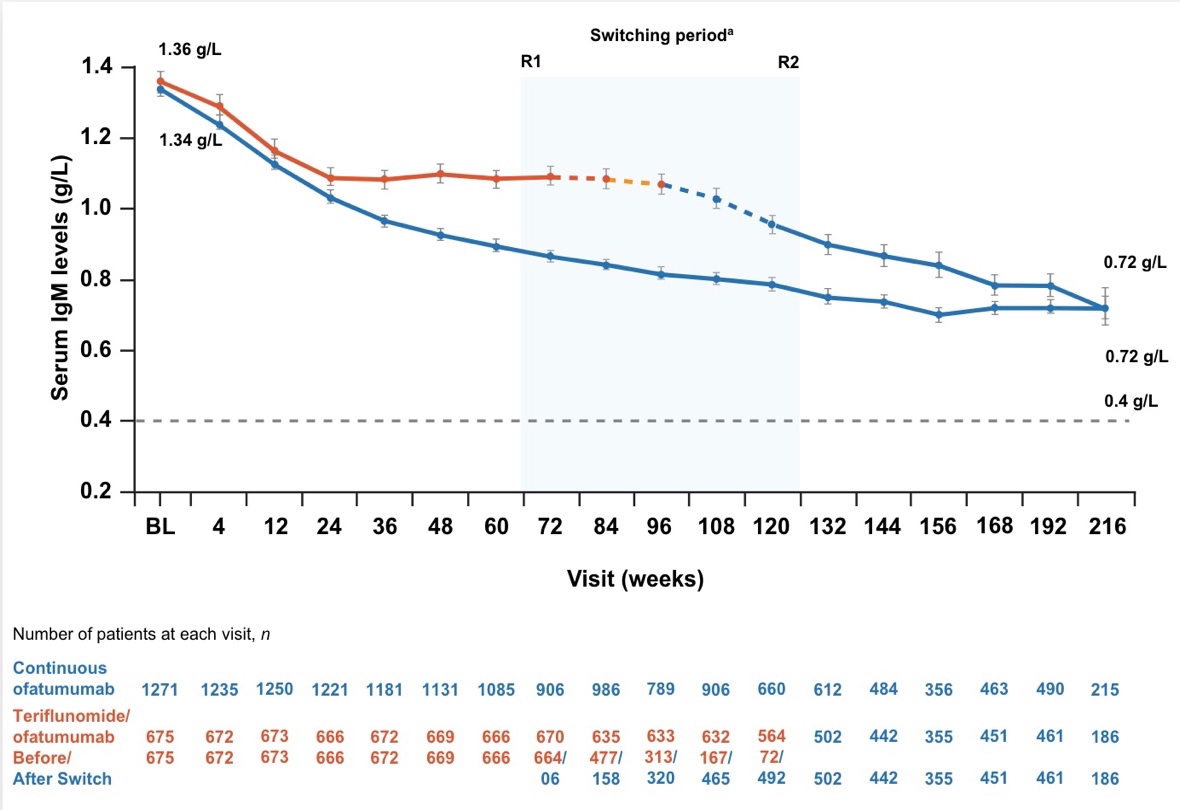


**(C)**

**
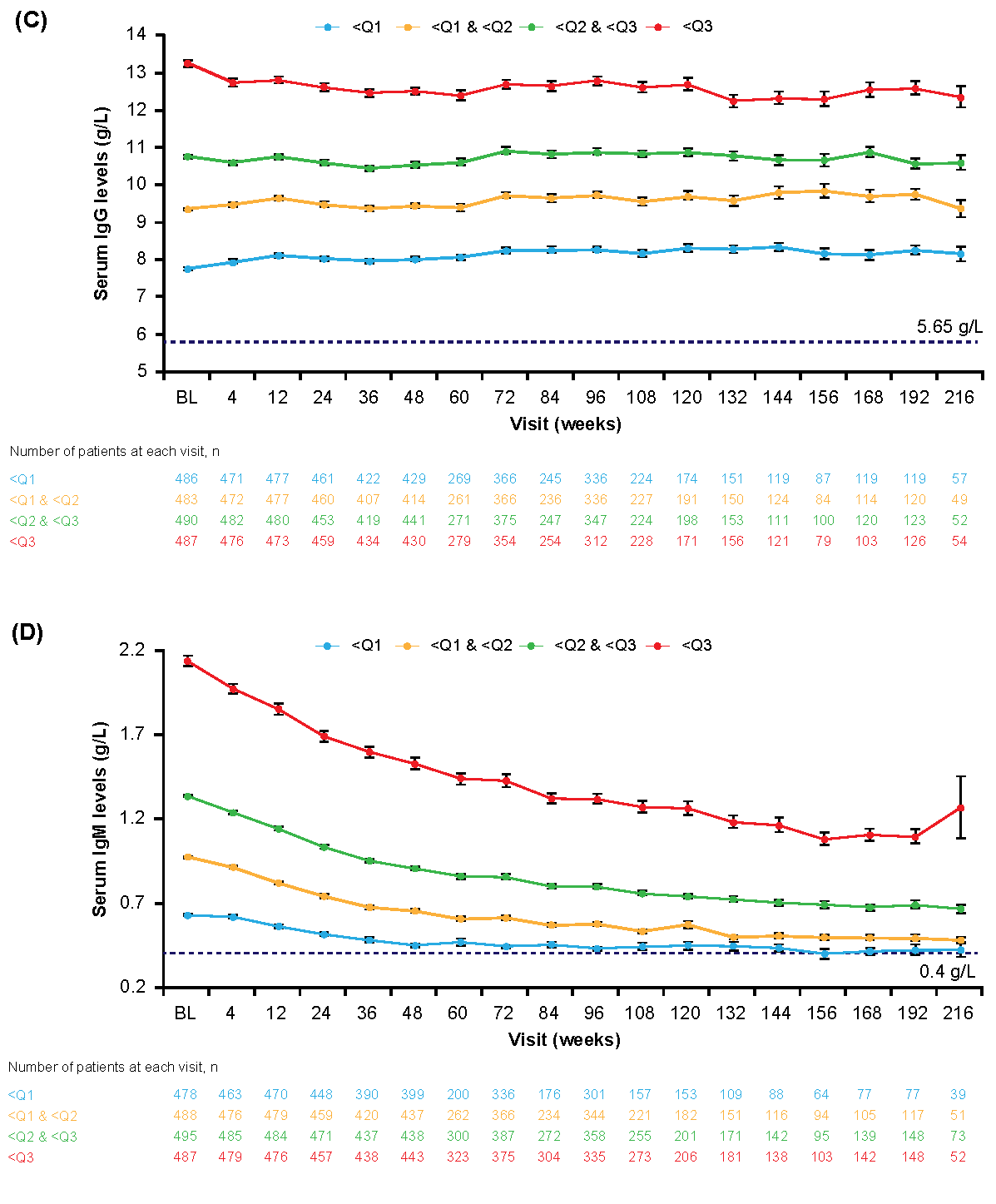
**

**(E)**


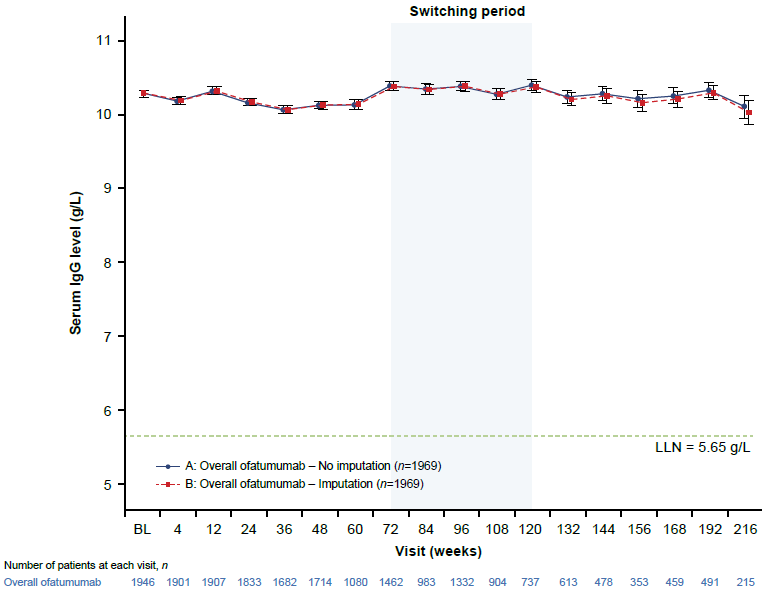


**(F)**


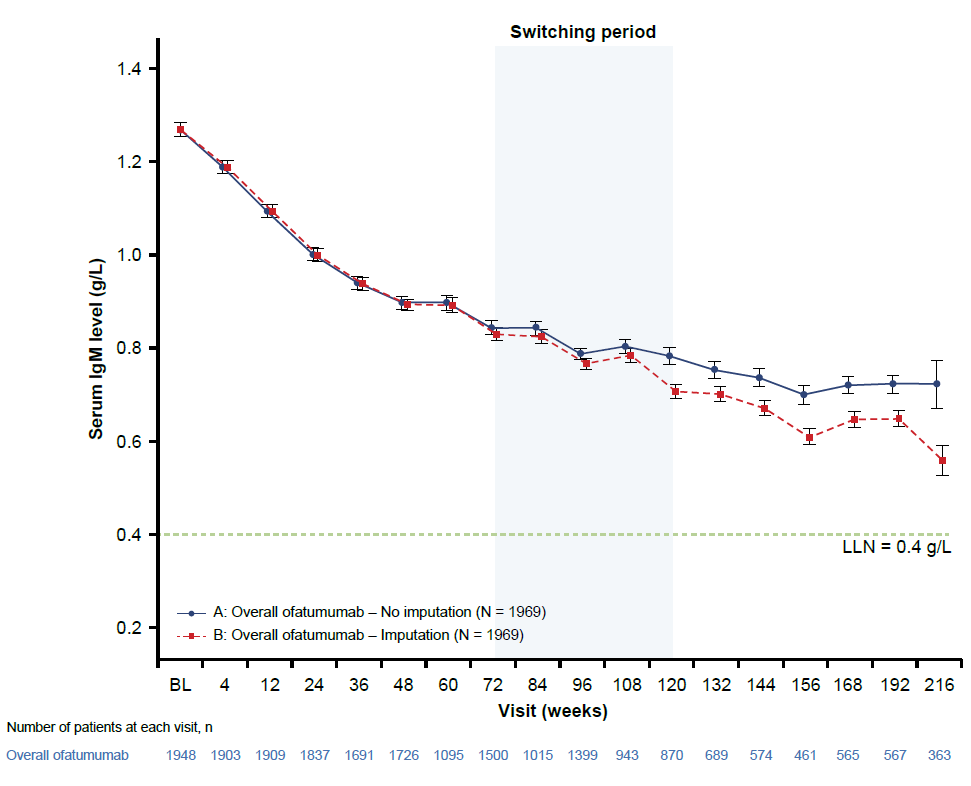


**(G)**


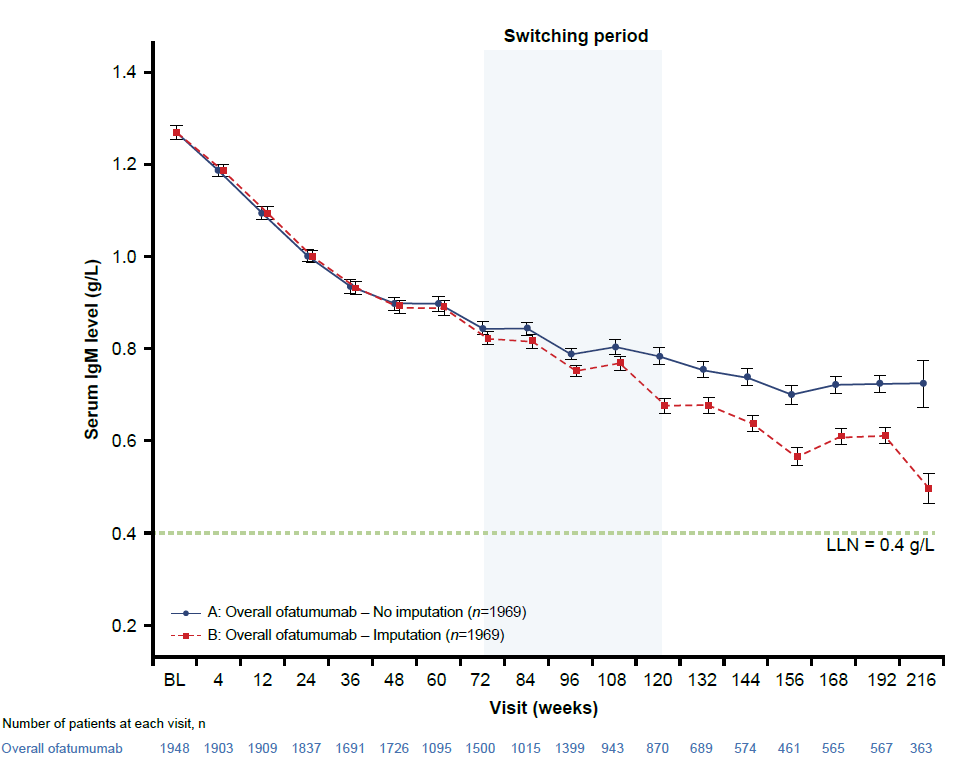


Data from the safety analysis set. Figures shown mean date; error bars represent ± standard error of the mean. Panels E and F: ± imputation (last observation carried forward) of data missing since IgG-/IgM-related treatment interruption; panel G: ± imputation by half LLN of data missing since IgM-related treatment interruption.

^a^Switching period refers to the patients started with teriflunomide and not applicable to the patients with ofatumumab in core period; For teriflunomide/ofatumumab group, data from 1st dose of teriflunomide until last dose of ofatumumab plus 100 days/analyses cut-off date have been used; R1: The first patient with first treatment emergent assessment in ofatumumab period after switching to ofatumumab (72 weeks); R2: The last patient with last treatment emergent assessment in teriflunomide period before switching to ofatumumab (120 weeks); For all pooled analyses, a fixed value of LLN (using ALITHIOS study reference) was used: IgG: 5.65 g/L and IgM: 0.4 g/L.

Quartiles for IgG (g/L): Q1, 8.57; Q2, 10.07; Q3, 11.51; Quartiles for IgM (g/L): Q1, 0.81; Q2, 1.14; Q3, 1.57.
*Data based on the overall safety population with only data from the first dose of ofatumumab included.

BL: baseline; Ig: immunoglobulin; LLN: lower limit of normal; SE: standard error of the mean.

## Supplementary Figure 6. (A) Lymphocyte levels with ≤4 years of ofatumumab treatment; (B) Neutrophil levels with ≤4 years of ofatumumab treatment (safety analysis set)

**(A)**

**
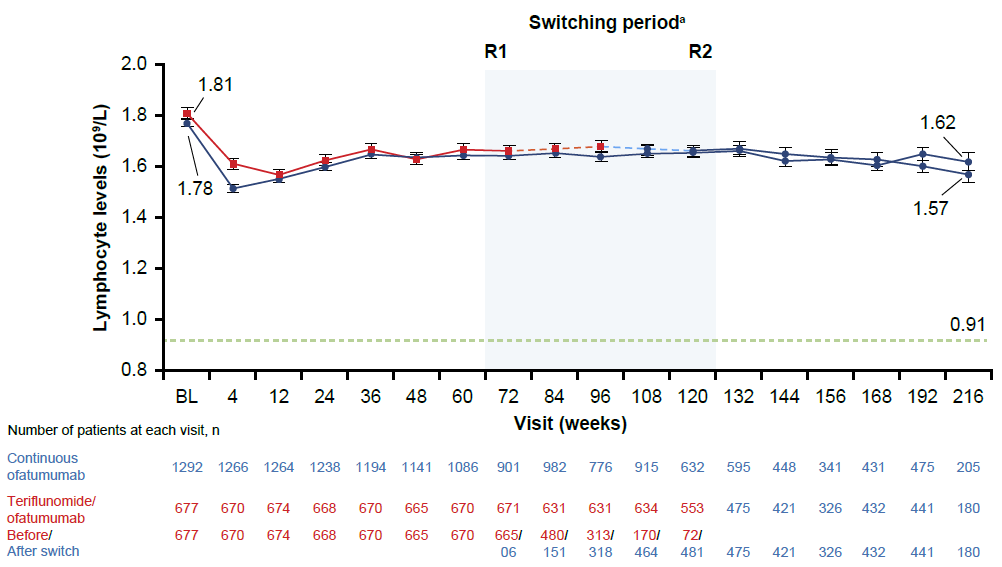
**

**(B)**

**
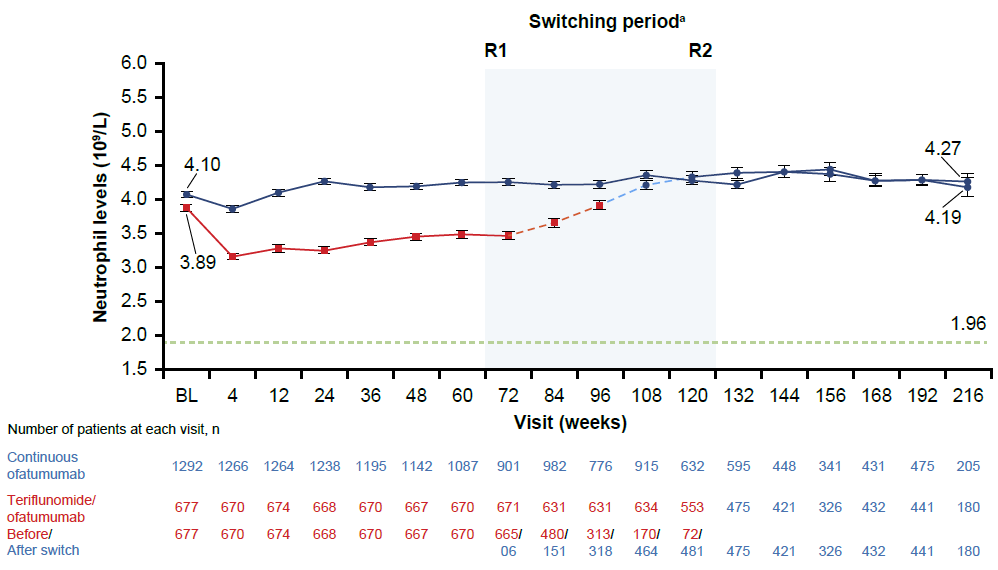
**

Data from the safety analysis set. ^a^Switching period refers to the patients started with teriflunomide and not applicable to the patients with ofatumumab in core period; for the teriflunomide/ofatumumab group, data are from the first dose of teriflunomide until last dose of ofatumumab plus 100 days/analyses cut-off date have been used (R1: the first patient with first treatment emergent assessment in ofatumumab period after switching to ofatumumab [72 weeks]; R2: the last patient with last treatment emergent assessment in teriflunomide period before switching to ofatumumab [120 weeks]).

BL: baseline; LLN: lower limit of normal; SE: standard error of the mean.
